# *De novo* transcriptome analysis of *Dysoxylum binectariferum* to unravel the biosynthesis of pharmaceutically relevant specialized metabolites

**DOI:** 10.3389/fpls.2023.1098987

**Published:** 2023-08-09

**Authors:** Patel Mohana Kumara, Eranna Varun, Joshi Renuka Sanjay, Anchedoddi Hanumegowda Madhushree, Ramesha Thimmappa

**Affiliations:** ^1^ Department of Biotechnology and Crop Improvement, Kittur Rani Chennamma College of Horticulture, Arabhavi, University of Horticultural Sciences, Bagalkot, Karnataka, India; ^2^ Center for Ayurveda Biology and Holistic Nutrition, The University of Trans-Disciplinary Health Sciences and Technology (TDU), Bengaluru, Karnataka, India; ^3^ Amity Institute of Genome Engineering, Amity University Uttar Pradesh, Noida, India

**Keywords:** *Dysoxylum binectariferum*, *de novo* transcriptome, rohitukine, chromone alkaloids biosynthesis, chalcone synthases, polyketide synthases

## Abstract

The tropical tree, *D. binectariferum*, is a prominent source of chromone alkaloid rohitukine, which is used in the semi-syntheses of anticancer molecules such as flavopiridol and P-276-00. The biosynthetic pathway of rohitukine or its derivatives is currently unknown in plants. Here, we explored chromone alkaloid biosynthesis in *D. binectariferum* through targeted transcriptome sequencing. Illumina sequencing of leaves and roots of a year-old *D. binectariferum* seedling generated, 42.43 and 38.74 million paired-end short reads, respectively. Quality filtering and *de novo* assembly of the transcriptome generated 274,970 contigs and 126,788 unigenes with an N50 contig length of 1560 bp. The assembly generated 117,619 translated unigene protein sequences and 51,598 non-redundant sequences. Nearly 80% of these non-redundant sequences were annotated to publicly available protein and nucleotide databases, suggesting the completeness and effectiveness of the transcriptome assembly. Using the assembly, we identified a chalcone synthase (CHS) and three type III polyketide synthases (PKS-III; non-CHS type) that are likely to be involved in the biosynthesis of chromone ring/noreugenin moiety of rohitukine. We also identified key enzymes like lysine decarboxylase in the piperidine pathway that make the piperidine moiety of rohitukine. Besides these, the upstream enzymes in flavonoid biosynthesis like phenylalanine ammonia-lyase (PAL), trans-cinnamate 4-hydroxylase (C4H),4-coumarate-CoA ligase (4CL), and chalcone isomerase (CHI) have also been identified. Also, terpene synthases that are likely to be involved in the biosynthesis of various terpenoid scaffolds have been identified. Together, the *D. binectariferum* transcriptome resource forms a basis for further exploration of biosynthetic pathways of these valuable compounds through functional validation of the candidate genes and metabolic engineering in heterologous hosts. Additionally, the transcriptome dataset generated will serve as an important resource for research on functional genomics and enzyme discovery in *D. binectariferum* and comparative analysis with other Meliaceae family members.

## 1 Introduction

Rohitukine, a prominent chromone alkaloid currently known to occur in five plant species belonging to *Meliaceae* and *Rubiaceae* families (Khadem and Marles, 2012; Varun et al., 2023). Rohitukine is a unique chromone alkaloid having a noreugenin chromone scaffold conjugated to a ring containing one or more nitrogen atoms (Houghton, 2002; Mohanakumara et al., 2010; Mohana Kumara, 2012). Flavopiridol (Sanofi) and P-276-00 (Piramal) two semi-synthetic derivatives of rohitukine are in the advanced stages of clinical trials for various cancer treatments (Jain et al., 2012). Flavopiridol (alvocidib; L868275; HMR-1275; NSC 649890 of Sanofi-Aventis + NCI) is an established cyclin-dependent kinases (CDK) inhibitor with broad specificity to CDK1, CDK2, and CDK4 leading to cell cycle arrest at both G1 and G2 phases (Sedlacek et al., 1996; Stadler et al., 2000; Łukasik et al., 2021). Flavopiridol is also a promising agent in inducing p53-independent apoptosis in Chronic Lymphocytic Leukaemia (CLL) and therefore this has been approved as an orphan drug for treating CLL (Christian et al., 2009; Albert et al., 2014; Mandal et al., 2021). Whereas P-276-00 is currently in phase II clinical studies for advanced refractory neoplasms and multiple myeloma (Christian et al., 2009; Borowczak et al., 2022). In addition to cancer, flavopiridol has also been shown to be effective in the treatment of arthritis and atherosclerotic plaque formation (Sekine et al., 2008; Chen et al., 2021).

Rohitukine was first reported in *Amoora rohituka* and later in *Dysoxylum binectariferum*, *Dysoxylum acutangulum* (*Meliaceae*), *Schumanniophyton magnificum* and *S. problematicum* (*Rubiaceae*) (Harmon et al., 1979; Naik et al., 1988; Ismail et al., 2009; Mohanakumara et al., 2010). Among these species, *D. binectariferum* accumulates the highest amount of rohitukine in stem bark (3-7% by dry weight). Whereas the closest relative of *D. binectariferum, D. malabaricum* does not accumulate rohitukine (Houghton, 2002; Mohanakumara et al., 2010). Also, various rohitukine derivatives such as dysoline, schumaniofioside A and chrotacumines have been reported from *D. binectariferum* (Ismail et al., 2009; Izwan Mohd Lazim et al., 2013; Morita et al., 2014; Mohana Kumara et al., 2016). Besides plants, endophytic fungi associated with *A. rohituka* and *D. binectariferum* have also been shown to produce rohitukine in culture (Mohana Kumara, 2012; Mohana Kumara et al., 2012; Kumara et al., 2014). But the biosynthetic pathway of chromone alkaloids in general has not been elucidated so far (Abe et al., 2005; Morita et al., 2007; Izwan Mohd Lazim et al., 2013). Earlier, desorption electrospray ionization mass spectrometry imaging (DESI-MSI) shows that rohitukine in germinating seedlings is largely restricted to the cotyledonary tissue, followed by the embryo and the seed coat (Mohana Kumara et al., 2015; Mohana Kumara et al., 2016; Varun et al., 2023). Within seedlings, rohitukine was predominantly distributed in the roots, collar region of the stem, and young leaves. In the stem and roots, rohitukine was primarily restricted to the cortex region (Mohana Kumara et al., 2016). DESI-MSI and electrospray ionization (ESI) tandem mass spectrometry (MS/MS) analysis revealed the presence of oxidized, acetylated glycosylated, and methylated derivatives of rohitukine (Mohana Kumara et al., 2015; Mohana Kumara et al., 2016). In addition to chromone alkaloids, *Dysoxylum* is also known to contain as many as 279 triterpenoids belonging to different scaffolds like dammarane, nortriterpenoid, oleanane, lupane, tirucallane, lanostane, cycloartane, glabretal and cyclopropane types (Yan et al., 2021; Naini et al., 2022). With recent advancements in sequencing technologies like genome and transcriptome sequencing of medicinal plants has become an important tool in understanding the biosynthetic pathway of metabolites of therapeutic relevance. For example, the genomes and transcriptomes of medicinal plants such as *Asparagus racemosus*, *Curcuma longa*, *Polygonum cuspidatum*, *Ocimum* spp., and *Azadirachta indica* have helped in establishing the different metabolic pathways (Narnoliya et al., 2014; Rajakani et al., 2014; Krishnan et al., 2016; Pandreka et al., 2021; Joudaki et al., 2023). These sequence resources form a base for further elucidation and functional characterization of the constituent metabolic pathways facilitating metabolic engineering in heterologous systems (Ma et al., 2021; Hu et al., 2023; Kwan et al., 2023). In the current study, we report the *de novo* transcriptome sequencing, assembly of the leaf and root tissues of *D. binectariferum* and annotation of genes in specialized metabolic pathways including chromones, alkaloids, flavonoids, sesquiterpenes and triterpene pathways. We also report differentially expressed genes in leaf and root tissues and study their tissue-specific gene expression. Finally, we identified potential genes involved in the above biosynthetic pathways and showed relative expression of their transcripts in leaves and roots.

## 2 Materials and methods

### 2.1 Plant material

**Figure 1:**
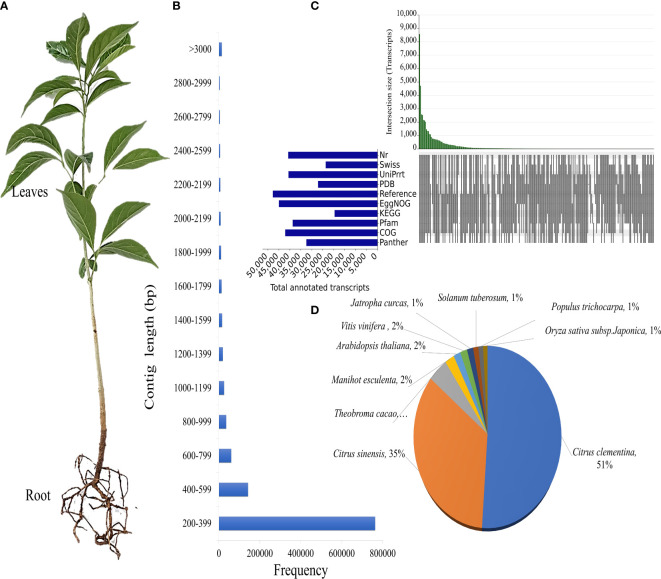
Annotation of *D. binectariferum* seedling transcriptome. **(A)** One-year-old *D. binectariferum* seedling used in transcriptome sequencing and metabolite analysis. **(B)** The contig’s length distribution of *de novo* assembled transcripts. **(C)** Upset plot showing the number of assembled unigenes, annotated to Nr, Swiss, TrEMBL/UniProt, PDB, Reference sequence, EggNOG, KEGG and Pfam databases, **(D)** Distribution of top hits (species) for the non-redundant unigenes identified in the transcriptome.

*D. binectariferum* was identified, collected and the voucher specimen was deposited at The University of Transdisciplinary Health Sciences and Technology herbarium, Bangalore (voucher specimen number; 122951-55). During the fruiting season *D. binectariferum* seeds were collected from Jog, Central Western Ghats, India (14^0^ 13’ 65” N and 74^0^ 48’ 35” E). Seeds were sown in polybags and seedlings were kept under shade with continuous watering and maintained in a nursery at the University of Transdisciplinary Health Sciences and Technology, Bengaluru. The leaves and roots of one-year-old seedlings of similar age and size were used in transcriptome sequencing and metabolite analysis (**Figure 1A**). The sampling was non-invasive with no impact on the natural growth or regeneration of *D*. *binectariferum* populations in the wild. And the study was conducted following relevant national and institutional guidelines.

### 2.2 RNA isolation from *D. binectariferum* and differential expression analysis

Total RNA was isolated from the root and leaf tissues of one-year-old *D. binectariferum* seedlings using TRIZOL reagent (Sigma Life Science, USA) (Meng and Feldman, 2010). Each sample included three biological replicates. The quantity and quality of total RNA was determined by NanoDdrop (Thermo Scientific) and agarose gel electrophoresis. The purity of total RNA was estimated using the absorbance ratio at 260/280 and 260/230, and the RNA integrity number (RIN). Samples showing acceptable RNA integrity numbers above 7 were used in library preparation. Sequencing was done from both 5’ and 3’ ends on the Illumina platform (NovaSeq 6000) according to the manufacturer’s instructions (Illumina Inc., San Diego, CA, USA).

About 500ng of total RNA was used in first-strand cDNA synthesis using the Takara cDNA synthesis kit according to the manufacturer’s instructions. qRT PCR analysis was carried out in triplicates using SYBR Green Universal Master Mix (Takara) in 98-well optical plates using Applied Biosystems, Quantum studio 3 Real-time qPCR system. Each (10μl) reaction contained a 10ng (2μL) cDNA template, 0.4µl of 5 pM each primer, and 5μl SYBR Green mix. Cycling conditions were as follows: 1 cycle of 50°C for 2 min, 95°C for 3 min, 40 cycles of 95°C for 10 sec, 55°C for 30 sec and 1 cycle of 95°C for 15 sec, 55°C for 1 min and 95°C for 15 sec. The EF2 gene (elongation factor 2) was used as a normalization control, and all samples were analysed in triplicates, and a dissociation curve validated the specificity of each primer pair (Xu et al., 2011; Moraes et al., 2015; Linardić and Braybrook, 2021; Xu et al., 2023). Relative quantification for levels of transcripts between the samples was calculated using 2−ΔΔCT method.

### 2.3 *De novo* transcriptome assembly

*D. binectariderum* roots and leaves (three biological replicates) were sequenced. The raw data obtained was quality checked by trimming and removing adaptor sequences and other low-quality sequences using the FastQC tool (http://www.bioinformatics.babraham.ac.uk/projects/). The raw reads were also processed using Trimmomatic v0.38.2 to remove low-quality reads using default parameters (Bolger et al., 2014). The clean reads were assembled using Trinity Version 2.9.1(Grabherr et al., 2011) with default parameters. The level of completeness of the final transcript assembly was evaluated using BUSCO v 5.4.4 tool (Seppey et al., 2019). Coding regions of the assembled transcripts were predicted using Transdecoder Version 5.5.0 (Haas et al., 2013). We removed redundant sequences, identified non-redundant or representative protein sequences using CD-HIT version 1.2, and retained the longest sequence with a minimum sequence identity threshold of 0.9 contigs in each cluster (Fu et al., 2012). Non-redundant or representative sequences (>200 amino acids cut off) were annotated based on sequence similarity using blastp against the following databases; NCBI non-redundant (Nr), Swiss-Prot 2018, TrEMBL/UniProt, Protein database, Reference sequence database with e value  10^− 3^ (NCBI BLAST+ blastp Galaxy Version 2.10.1+galaxy2; Christiam Camacho et al., 2009; Peter et al., 2015), HMMER/Protein family (Pfam) v3.3.2 (https://www.ebi.ac.uk/Tools/hmmer/), EggNOG (http://eggnog5.embl.de/#/app/home), Clusters of Orthologous Groups of proteins (COGs), Gene Ontology (GO)(http://www.pantherdb.org/), and Kyoto Encyclopaedia of Genes and Genomes (KEGG) (https://www.kegg.jp/) (**Figure S1**).

### 2.4 Differential expression analysis

Global differentially expressed genes (DEGs) analysis between root and leaf samples was performed using DESeq2 (ver 2.11.40.7) tool with the Benjamini-Hochberg procedure (Love et al., 2014). The expression levels were calculated and normalized using TPM methods (Lin et al., 2016; Patro et al., 2017). DEGs were identified with adjusted FDR ≤ 0.05 (false discovery rate), log2 (fold change) of > 2 and FPKM value of >11. An online enrichment tool, ShinyGO v0.75, was used to identify different KEGG pathways enriched in DEGs (http://bioinformatics.sdstate.edu/go75/). The significance of KEGG terms was determined by a p-value or q-value of ≤0.05. Further investigation was performed with selected DEGs. Within this list, we concentrated on genes associated with 17 different metabolic pathways comprising terpenoids, flavonoids, piperidine, and chromone alkaloids etc.

### 2.5 Extraction of rohitukine and quantification

Metabolites were extracted from the leaf and root tissues of *D. binectariferum* using methods described earlier (Mohanakumara et al., 2010). Briefly, the freeze-dried samples were ground to a fine powder. Extraction was carried out using methanol (10 mL). The extracts were vortexed, sonicated (30 min) and centrifuged (8,000 rpm for 10 min). Next, extracts were passed through membrane filters (0.2 µm) and kept in airtight vials at -20°C until further use. Samples were analyzed using reverse-phase HPLC (Shimadzu, LC20AT, Japan), RP-18 column (4.6 x 250 mm, 5μm) with UV absorbance at 254 nm. The standard rohitukine was prepared with a series of concentrations (0.2 – 1.0 mg/ml) using liquid chromatography-mass spectrometry (LC-MS) grade methanol and filtered using 0.2µ syringe filters. Acetonitrile and 0.1% TFA were used in gradient mode as the mobile phase. The linear graph obtained (y = mx) was used in quantification of rohitukine in samples (R^2 ^= 0.99) (Mohana Kumara et al., 2016). The significance of rohitukine content in the leaf and root of *D. binectariferum* was tested using t-tests (unpaired), F-tests, and Kruskal-Wallis tests using Past 4.11 (Hammer et al., 2001).

### 2.6 GC-MS analysis

Leaf and root tissues of *D. binectariferum* were also subjected to volatile analysis using GC-MS (Sharma et al., 2021). Leaf and root samples were dried at 40 °C for 8-12 hrs in a hot air oven and 1.0 ± 0.01 g of fine powder was subjected to headspace analysis using GC-MS fitted with RTx-volatiles capillary column (3.0 m × 0.25 mm × 0.25 μm). The analysis was done using a Shimadzu^®^ - Nexis GCMS 2030 coupled to a mass spectrometer with a triple quadrupole TQ8040NX, equipped with an HS-20 auto-sampler (Shimadzu, Tokyo, Japan). The following GC temperature program was used; the column oven temperature was maintained at 80°C for 1 min., followed by two heating ramps of 5 and 10°C/min. until reaching temperatures of 150 °C and 200 °C, respectively. Mass spectra were obtained using electron impact at 70 eV and a start and end mass-to-charge ratio (*m/z*) of 30 and 500, respectively. The compounds were identified by comparison to the mass spectra from library databases (NIST 98; http://www.nist.gov) and by calculating Kovat’s indices using alkane standards (C8-C24) RT values.

## 3 Results and discussion

### 3.1 *De novo* assembly

The *D. binectariferum* transcriptome generated 42.43 and 38.74 million paired-end short reads (150 bp) for leaves and roots, respectively. Filtering for quality resulted in 41.52 (97.85%) and 38.09 (98.33%) million clean reads for the leaf and roots, respectively (**Table 1**). *De novo* assembly of the short reads generated 274,970 contigs and 126,788 unigenes from the whole transcriptome with an N50 length of 1,560 bp (**Table 2**). The average GC content of the contigs derived from the transcriptome was 42.6% (**Table S1**). Of the 2,326 BUSCOs in the Eudicots dataset, 2,142 (92.1%) complete BUSCOs were detected in the assembly (**Table S2**). The results indicated that the assembly was almost complete with an adequate representation of the gene directory. From the assembly, 67.67% of the contigs (763,242) were 200-400 bp in length, 21.0% (237,930) were 400-1,000 bp, 7.43% (83,771) were 1,000-2,000 bp, 2.55% (28,784) were 2,000-3,000 bp, and only 1.26% (14,233) exceeded 3,000 bp (**Figure 1B**). Using the CD-HIT tool (>200 amino acids cut off), we identified 51,598 nonredundant protein sequences from a total of 117,619 translated unigenes/protein sequences. After removing the redundancy, *De novo* assembly generated short reads of 117,619 contigs and 51,340 unigenes from the whole transcriptome with an N50 length of 1,176bp. The average GC content of the contigs derived from the transcriptome was 46.89% (**Table S1**) (**Table 2**).

**Table 1:** Quality analysis of *Dysoxylum binectariferum* transcriptome.

Samples	Replicates	Forward R1/Reverse R2	Number of seq. Before trimming	Number of seq. After trimming	GB Before trimming	GB After trimming
DBR (root)	DBR1	R1	43,318,058	42,370,596	14.5	13.7
		R2	43,318,058	42,370,596	14.5	13.7
	DBR2	R1	43,000,082	41,776,589	14.4	13.5
		R2	43,000,082	41,776,589	14.4	13.4
	DBR3	R1	40,989,199	40,431,285	13.8	12.9
		R2	40,989,199	40,431,285	13.8	12.8
Avg.			42,435,779	41,526,156	14.2	13.3
DBL (leaf)	DBL1	R1	45,090,230	44,321,743	15.2	14
		R2	45,090,230	44,321,743	15.2	13.7
	DBL2	R1	39,422,787	38,839,563	13.2	12.3
		R2	39,422,787	38,839,563	13.2	12.2
	DBL3	R1	31,723,969	31,134,812	10.7	9.6
		R2	31,723,969	31,134,812	10.7	9.4
Avg.			38,745,662	38,098,706	13.0	11.9

**Table 2:** Summary of *D. binectariferum* transcriptome final assembly.

Assembly	Contigs/Unigenes
# contigs	117,619
# contigs (>= 0 bp)	117,619
# contigs (>= 1000 bp)	51,340
Largest contig	15,318
Total length	132,769,470
Total length (>= 0 bp)	132,769,470
Total length (>= 1000 bp)	82,035,717
N50	1,176
N90	687
auN	1,481.2
L50	36,872
L90	96,885
GC (%)	46.89
CD-HIT PROTEIN (>200)	51,598

### 3.2 Functional annotation

To identify putative protein functions, all the assembled unigenes were annotated using the Basic Local Alignment Search Tool (BLAST) against ten publicly available protein databases. Out of 51,598 unigenes, 40,699 (78.88%) were annotated to Nr, 23,618 (45.77%) to Swiss-Prot, 40,552 (78.59%) to TrEMBL/UniProt, 27,101 (52.52%) to Protein database, 47,675 (92.4%) to a Reference sequence database, 44,919 (87.06%) to EggNOG, 19548 (37.89%) to KEGG, 38,297 (74.22%) to Pfam, 42,040 (81.48%) to COG and 36,530 (70.8%) to Panther GO (**Figure 1C**; **Additional File 1**). *Citrus clementina* (7,174), *Citrus sinensis* (4,837), *Theobroma cacao* (621), *Manihot esculenta* (296), and *A. thaliana* (224) contributed to the most gene annotations (**Figure 1D**). Two *Citrus* species were the major hits because both *Citrus* and *D. binectariferum* are related phylogenetically as well as encode shared metabolic pathways (Levitsky and Dembitsky, 2014; Bhambhani et al., 2017; Du et al., 2021; Hou et al., 2022).

**Figure 2:**
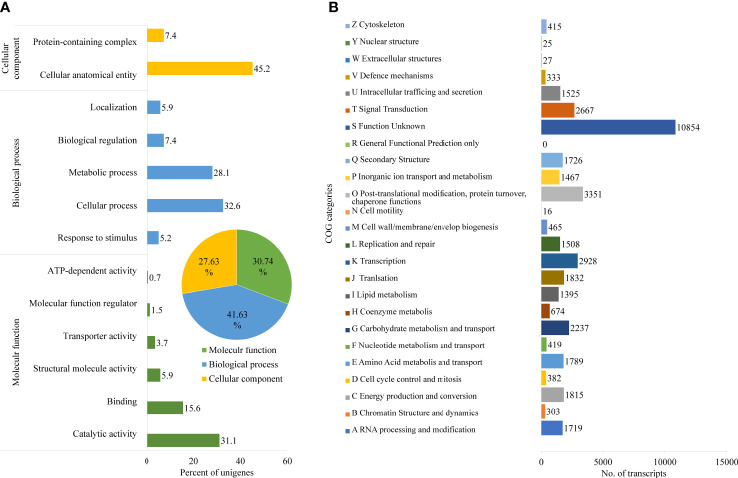
Functional annotation of *D. binectariferum* transcriptome based on Gene Ontology (GO). **(A)** GO functional classifications of assembled *D. binectariferum* unigenes. Insert in pie chart showing percentage of annotation to three different classes of gene ontology. **(B)** Clusters of Orthologous Groups (COG) functional classifications of assembled *D. binectariferum* unigenes and associated number of transcripts with COG function categories.

Further, Nr unigenes were annotated to three major ontologies (GO): molecular function (MF), biological process (BP), and cellular component (CC). BP comprises 41.63% of the total assigned annotations, whereas CC and MF comprised 27.63% and 30.74% respectively. Among biological processes, the unigenes are predominantly annotated to the cellular process (32.6%) and metabolic process (28.1%), followed by biological regulation (7.4%). Similarly, for the CC category, the largest number of unigenes were assigned to cellular anatomical entities (45.2%) and protein-containing complexes (7.4%). While, in the MF category, the catalytic activity (31.1%), and binding (15.6%) were the most annotated (**Figure 2A**). A total of 42,040 transcripts were assigned to 25 COG classifications with the majority in the category “function unknown” (10,854, 21.04%), followed by “post-translational modification, protein turnover, chaperone functions” (3,351, 6.49%), “transcription” (2,928, 5.67%) and “carbohydrate metabolism and transport” (2,237, 4.34%) (**Figure 2B**).

**Figure 3:**
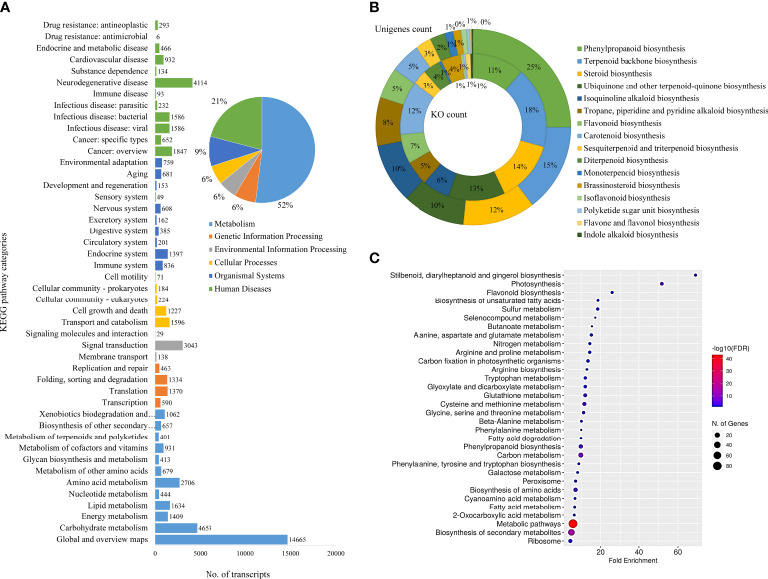
Functional annotation of *D. binectariferum* transcriptome based on KEGG. **(A)** Assembled *D. binectariferum* unigenes annotated to the functional classification of the Kyoto Encyclopedia of Genes and Genomes (KEGG) database and their associated number of transcripts are shown for each of the KEGG functional category. **(B)** Pie chart showing the selected KEGG pathways related to specialized metabolic pathways and the inner ring represents the KO category while the outer ring represents its respective unigenes count, **(C)** KEGG pathway enrichment from final annotated transcripts of *D. binectariferum*. The vertical axis represents the pathway’s name, and the horizontal axis represents the fold enrichment. The size and color of bubbles indicate the number and degree of enrichment of different metabolites, respectively.

The KEGG classifications for the assembled unigenes were used to evaluate the completeness of the transcriptome library as well as the effectiveness of the annotation process for identifying the specialized metabolic pathways. A total of 19,548 assembled unigenes were assigned into six main functional categories (Metabolism, Genetic Information Processing, Environmental Information Processing, Cellular Processes, Organismal Systems, and Human Diseases) and 46 subcategories (**Figure 3A**) and 431 KEGG pathways. The two most abundant sub-categories were “metabolism” and “human diseases”, covering 57.47% and 23.14% of the total annotations, respectively. The rest were covered by the remaining categories of Genetic Information Processing (7.28%), Environmental Information Processing (6.22%), Cellular Processes (6.39%) and Organismal Systems (10.13%). Furthermore, the unigenes coding for specialized metabolite biosynthesis were analyzed. The 17 major specialized metabolic pathways were selected and their respective KO and unigene counts are shown in **Figure 3B** and **Additional File 2**). Of these, 213 unigenes were assigned to “Phenylpropanoid biosynthesis”, followed by 124 unigenes for “Terpenoid backbone biosynthesis”, 102 for steroid biosynthesis, and others. These annotations form a basis for the functional characterization of genes involved in the specialized metabolism and regulation of *D. binectariferum* (Kanehisa and Goto, 2000; Liu et al., 2013; Bhambhani et al., 2017).

### 3.3 Metabolic pathway analysis

We identified 6,495 DEGs in total, including 3,532 genes that were upregulated in roots and 2,963 genes that were downregulated in leaves. Further, unigenes related to 17 different specialized metabolic pathways were analyzed for their expression levels (log_2_fold) in roots and leaves. Of the 736 DEGs that were found to be involved in specialized metabolism, 284 of them were upregulated and 452 of them were downregulated in the root compared to the leaf (Additional file 3 and 4). Based on KEGG pathway enrichment of the bubble diagram, carbon metabolism, stilbenoid, flavonoid, unsaturated fatty acids, and phenylpropanoid biosynthesis were the most dominant pathways, and the majority of KEGG-identified genes were associated with metabolic pathways and secondary metabolite biosynthesis (**Figure 3C**).

#### 3.3.1 Identification of unigenes involved in the terpenoid pathway

**Figure 4:**
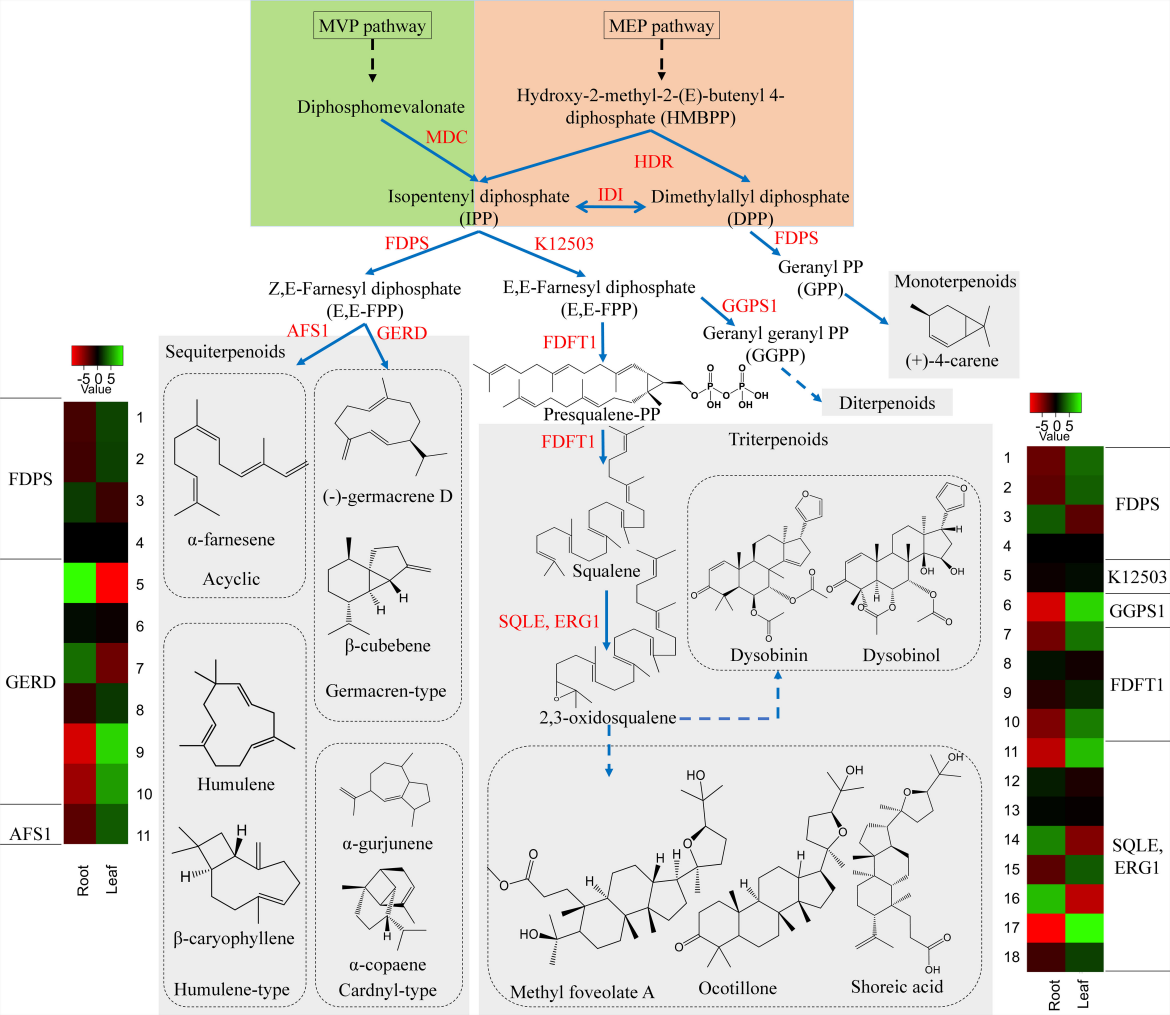
*D. binectariferum* candidates involved in the biosynthetic pathways of terpenoids. Key biosynthetic enzymes (red)) identified from the *D. binectariferum* transcriptome are shown at each step. Heat-map displaying differential expression of unigene transcripts (enzymes) of *D. binectariferum* transcriptome are shown for roots and leaves. Details of numbers labelled in the Heat-map are provided in the additional file 3. MDC-Mevalonate pyrophosphate decarboxylase; HDR- 4-Hydroxy-3-methylbut-2-enyl diphosphate reductase (HDR)/HMBPP reductase; IDI- Isopentenyl diphosphate isomerase; FDPS- Farnesyl diphosphate synthase; FDPS-Farnesyl diphosphate synthase; K12503: short-chain Z-isoprenyl diphosphate synthase; AFS 1-α-farnesene synthase; GERD- (-)-Germacrene D synthase; GGPS1-Geranylgeranyl pyrophosphate synthase1; SQLE/ERG1- Squalene epoxidase/Squalene monooxygenase.

Terpenoids comprise the largest group of structurally diverse natural compounds and are known to be biosynthesized *via* two biosynthetic routes; the 2-C-methyl-D-erythritol 4-phosphate (MEP) pathway and the mevalonic acid (MVA) pathway (Sandeep and Ghosh, 2020). The isoprene unit (C5) is synthesized from the MEP pathway and is engaged in the formation of mono-(C10), Di-(C20) and other terpenoids. Whereas the isoprene unit from the MVA pathway is used in the synthesis of triterpene (C30) and sesquiterpenes (C15) (Schilmiller et al., 2009; Zhao et al., 2013; Ghissing et al., 2023). In the *D. binectariferum* transcriptome, around 269 unigenes (70 key enzymes) were found to be associated with the terpenoid pathways (**Figure 4**; **Figure S2**). Of these, we identified 48 unigenes encoding 6 key enzymes in the mevalonate pathway (MVA) and 29 unigenes encoding 8 key enzymes in the MEP pathway leading to the formation of isopentenyl diphosphate (IPP) and dimethylallyl diphosphate (DMAPP) (Kuzuyama, 2002). IPP and DMAPP go through a series of condensation reactions by prenyl diphosphate synthases to form prenyl diphosphates like geranyl diphosphate (GPP; C10), farnesyl diphosphate (FPP; C15), geranylgeranyl diphosphate (GGPP; C20) and other diphosphates. These prenyl diphosphate precursors form a basis for the biosynthesis of a diverse classes of terpenoids currently observed in plants (**Figure 4**; **Figure S2**) (Lorigooini et al., 2020). The formation of FPP from IPP by the enzyme short-chain Z-isoprenyl diphosphate synthase (K12503, 1 unigene) leads to triterpenoid synthesis *via* squalene. The squalene is oxidized to form 2, 3 oxidosqualene by squalene monooxygenase (SQLE, 9 unigenes) and 2, 3 oxidosqualene is the central precursor in biosynthesis of diverse triterpenoids including dammarane, cabraleadiol, 3-epiocotillol, methyl shoreate, and others (Yan et al., 2014; Fan et al., 2021). The 2, 3 oxidosqualene also leads to the formation of sterols (phyto- and ergosterols) involved in sitosterol and phytosterols biosynthesis in plants. Farnesyl diphosphate synthase (FDPS, 4 unigenes) accepts both DMAPP and IPP from MEP and MVA pathways in the mono and sesquiterpenoids (Szkopińska and Płochocka, 2005). We analyzed the volatile components of the leaf and root tissues of *D. binectariferum* using GC-MS to identify the possible precursors of the terpenoids. The leaf contained 14% monoterpenes, 6.5% sesquiterpenes, 1.5% ketones, and 1.27% cycloalkanes, while the root contained 49.75% sesquiterpenes, 8.86% sesquiterpenoids, 0.2% aldehydes, and 0.11% ketones (**Table S3**). The farnesyl diphosphate synthase (FDPS, 4 unigene) is involved in the synthesis of the sesquiterpenoids. The enzyme (-)-germacrene D synthase (GERD, 6 unigenes) makes germacren-type sesquiterpenes. In GC-MS we detected several sesquiterpenenes including α- and β-cadinenes, α- and β-copaene α-ylangene, caryophyllene, alloaromadendrene, α-guaiene, and germacrene-D in leaves and α-muurolene in the root. Additionally, monoterpene (+)-4-carene was also detected in *D. binectariferum* leaves (**Table S3**; **Figure S3**). All these sesquiterpenes detected in GC-MS analysis were mapped to different terpenoid biosynthetic routes (**Figure 4**; **Figure S3**). The transcriptome showed 12 unigenes involved in monoterpenoid biosynthesis and 21 unigenes encoding 7 enzymes in gibberellin biosynthesis (2 beta-dioxygenase GA2ox, 7 unigenes, ent-kaurenoic acid monooxygenase KAO, 4 unigenes) (**Figures 3B**, **4**). In total, the MEP pathway predominates in the roots and MVA pathway in the leaf which contributes to the generation of many volatile molecules (Inaba and Ito-Inaba, 2010; Tomar et al., 2013; Pérez et al., 2022) and they play key roles in the biosynthesis of various defensive compounds (Yan et al., 2021; Naini et al., 2022; Ghissing et al., 2023).

#### 3.3.2 Identification of unigenes involved in the flavonoid pathway

**Figure 5:**
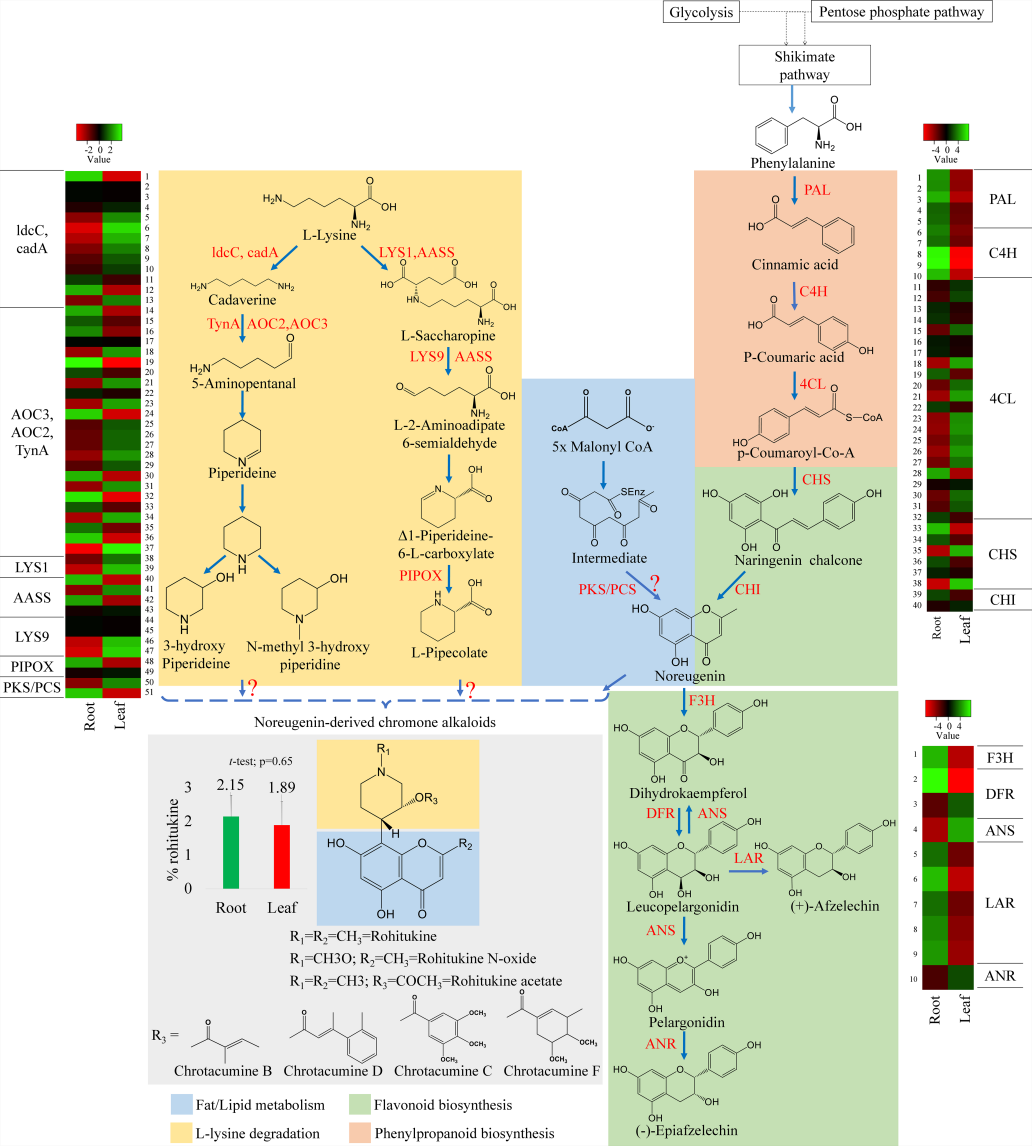
Putative biosynthetic pathway of rohitukine. Convergence of piperidine (also called lysine degradation) and shikimate routes (highlighted with different colours) in biosynthesis of chromone alkaloids in *D. binectariferum.* (rohitukine). Key biosynthetic enzymes (red) identified from the *D. binectariferum* transcriptome are shown at each. Steps highlighted with question marks denote unknown enzymatic conversions or candidate enzymes. Dotted arrows represent multiple steps or enzymes. Heat-map displaying differential expression of unigene transcripts (enzymes) *D. binectariferum* transcriptome are shown for roots and leaves. Details of numbers labelled in the Heat-map are provided in the additional file 3. The graph (insert) showing rohitukine content in the root and leaves of a one-year-old *D. binectariferum* seedling that was used in transcriptome sequencing. Mean (percent rohitukine, % of dry weight) and the error bars derived from three independent biological replicates (n =3). Piperidine (Lysine degradation) pathway; ldcC-Lysine decarboxylase; cadA- Lysine decarboxylase; LYS1- Lysine-forming saccharopine dehydrogenase; AASS- Alpha-aminoadipic semialdehyde synthase; AOC2/AOC3/TynA- primary-amine oxidase; LYS9- L-glutamate-forming saccharopine dehydrogenase 9; PIPOX- Sarcosine oxidase/L-pipecolate oxidase. Shikimate/Flavonoid/chromone alkaloid pathways: PAL- Phenylalanine ammonia-lyase; C4H- Trans-cinnamate 4-hydroxylase;4CL-4-coumarate-CoA ligase; PKS/PCS, Type III Polyketide synthase/Pentaketide chromone synthase; CHI, Chalcone isomerase; F3H, flavanone 3-hydroxylase; DFR, Dihydroflavonol 4-reductase/flavanone 4-reductase; LAR, Leucoanthocyanidin reductase; ANS, Anthocyanidin synthase; ANR, Anthocyanidin reductase.

In flavonoid biosynthesis, twelve key enzymes are involved in the conversion of *p*-coumaroyl CoA to naringenin, and *D. binectariferum* revealed 44 unigenes to be associated with all the 12 key enzymes (**Figure 3B**). Chalcone synthase (*CHS*, 6 unigenes) is the first key enzyme in flavonoid biosynthesis that converts 4-coumaroyl CoA to naringenin chalcone. Then the isomerization of naringenin chalcone to noreugenin is catalyzed by the enzyme chalcone isomerase (*CHI*, 2 unigenes). Noreugenin forms a central precursor from which all other flavonoids are derived (Liu et al., 2013). The naringenin is converted to dihydrokaempferol by an enzyme naringenin 3-dioxygenase (*F3H*, 1 unigene) and dihydrokaempfero is further converted to leucopelargonidin by a bifunctional enzyme dihydroflavonol 4-reductase/flavanone 4-reductase (*DFR*, 3 unigenes). Leucopelargonidin is a key molecule, where (+)-Afzelechin and pelargonidin are formed by the enzymes leucoanthocyanidin reductase (*LAR*, 4 unigenes) and anthocyanidin synthase (*ANS*, 1 unigene) respectively, and also converted back to the dihydrokaempferol by *ANS* (Zhao et al., 2017; Wang et al., 2021). Further, pelargonidin is reduced to form (-)-Epiafzelechin by an enzyme anthocyanidin reductase (*ANR*, 1 unigene) (**Figure 5**). The genes that encode these enzymes, *PAL* (3 unigenes), *C4H* (5 unigene), *CHS* (6 unigenes), *CHI* (2 unigene) and *4CL* (22 unigenes) were found to be significantly upregulated in roots compared to leaves (**Figure S4**).

**Figure 6:**
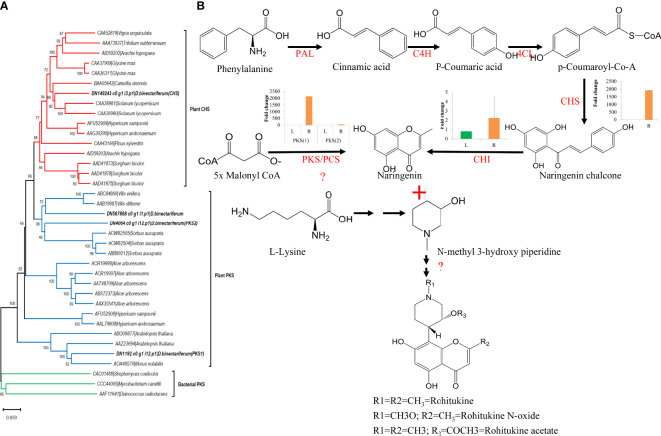
Candidate polyketide synthases identified from *D. binectariferum* transcriptome and their likely role in rohitukine/chromone alkaloids biosynthesis. **(A)** Phylogenetic tree of functionally characterized chalcone synthase (CHS) and polyketide synthase IIIs (PKSIII) from diverse plants along with CHS and PKS-III candidates identified from *D. binectariferum* transcriptome. Tree was created using MEGA7 with 1000 bootstrap values. Candidates highlighted in bold represent the candidates from *D. binectariferum*. **(B)** Predicted biosynthetic pathway of rohitukine/chromone alkaloids in *D. binectariferum.* Graphs in the inserts represent quantitative expression of key genes in the chromone alkaloid biosynthetic pathways (qRT mean ± SD, n=3, L; Leaves, R; Roots). PAL, Phenylalanine ammonia-lyase; C4H, Trans-cinnamate 4-hydroxylase; 4CL, 4-coumarate-CoA ligase; PKS/PCS, Type III Polyketide synthase/Pentaketide chromone synthase; CHI, Chalcone isomerase.

In parallel, using HPLC, we also measured rohitukine content in *D. binectariferum* leaf and the root tissues and the data showed that rohitukine content was comparatively more in root (2.15 ± 0.62%) than in the leaf (1.89 ± 0.69%) (**Figure 5**; p > 0.05; not significant). To test if the expression pattern of flavonoid and other associated pathway genes is correlated with rohitukine content we measured their expression patterns using quantitative Realtime PCR (qRT) in leaves and roots. The upstream genes *PAL, C4H, 4CL*, and *CHI* are involved in the biosynthesis of the key precursor naringenin (**Table S4**; **Figure S4**) and all these four genes were highly expressed in the roots compared to the leaves and this was comparable to DEseq-RNA-seq expression data (**Figure S4**). Further, using BLASTP with *Arabidopsis CHS* as a query sequence, we identified *CHS* like genes from *D. binectariferum* transcriptome. In total four full-length *CHS*-like genes were identified. Of these, one of them corresponds to the *CHS* (DN149243) and it is likely to be involved in the biosynthesis of noreugenin. The other three unigenes DN1192 (*PKS1*), DN4064 (*PKS2*), and DN567668 belong to type-III polyketide synthases (*PKS-III)* (**Figure 6A**; **Table S5**). We subjected these candidates for phylogenetic analysis along with other functionally characterized CHSs and PKS-IIIs from plants together with bacterial PKSs as out groups. These genes were grouped into three clusters; chalcone synthases (*CHS*), plant non *CHS/PKS-III*, and bacterial *PKS* (**Figure 6**; **Table S5**). *D. binectariferum PKS-III* candidates DN1192 (PKS1), DN4064 (PKS2), and DN567668 were clustered with known plant *PKS-III’s* and it is likely that one of these could be involved in the biosynthesis of chromone alkaloids (**Figure 6**). The candidates *PKS1* and *PKS2* are highly expressed in the root with low to negligible expression in the leaves of *D. binectariferum* (**Figure 6B**) corresponding roughly with the rohitukine content in roots. For example, a pentaketide chromone synthase (*PCS*) that makes noreugenin (5,7-dihydroxy-2-methylchromone) by successive condensation of five malonyl-CoA precursor units is known from the plant *Aloe arborescens* (Izwan Mohd Lazim et al., 2013). Therefore, functional characterization of the *PKS-III* like candidates from *D. binectariferum* likely reveal *PCS* like enzyme in the biosynthesis of rohitukine or chromone alkaloids.

#### 3.3.3 Identification of unigenes involved in the piperidine pathway

The L-lysine degradation pathway seem to provide N-containing phenol ring in chromone alkaloids. The amino acid L-lysine is degraded by a known enzyme lysine decarboxylase (*ldcC, cadA*, 13 unigenes) leading to the formation of cadaverine (Tomar et al., 2013). Next, primary-amine oxidase (*AOC3/AOC2/tynA*, 24 unigenes) converts cadaverine to 5-aminopentanal. Cadaverine and 5-aminopentanal are the central precursors in the biosynthesis of L-pipecolate and piperidine (Reimer et al., 2017) (**Figure 5**). The following enzymes are known to be involved in pipecolate and peridine biosynthesis from cadaverine; L-lysine-forming saccharopine dehydrogenase (*LYS1*, 2 unigenes), alpha-aminoadipic semialdehyde synthase (*AASS*, 5 unigenes), alpha-aminoadipic semialdehyde synthase, saccharopine dehydrogenase (*LYS9*, 3 unigenes) and sarcosine oxidase/L-pipecolate oxidase (*PIPOX*, 2 unigenes) (**Figure 5**) (Hartmann and Zeier, 2018). Presence of these candidates/unigenes and their expression in leaves suggest that the L-lysine degradation pathway is likely operative in *D. binectariferum* (**Figures 5**, **6B**). In plants with L-lysine degradation pathway the associated pathway genes are normally expressed in leaves and the pathway end products like pyridine and piperidine alkaloids are also present in leaves (defence related) (Inaba and Ito-Inaba, 2010; Khadem and Marles, 2012; Hartmann and Zeier, 2018).

#### 3.3.4 Putative chromone alkaloid biosynthetic pathway

Rohitukine is a chromone alkaloid consisting of noreugenin or flavone scaffold attached to a nitrogen containing piperidine ring (Harmon et al., 1979; Mohanakumara et al., 2010). Noreugenin chromone scaffold is a central precursor in biosynthesis of diverse chromone alkaloids including rohitukine and its derivatives. Noreugenin is made either through; a) a flavonoid pathway or b) through successive condensation of multiple malonyl co-A units by type-III polyketide synthase-like enzymes. The presence of the unigenes coding for PKS-III candidate enzymes as well as their high expression in roots where rohitukine is highly accumulated suggests that the route ‘b’ is more plausible (**Figure 6B**). And the piperidine ring is likely derived from the L-lysine degradation pathway and condensation of piperidine moiety and noreugenin yields rohitukine or chromone alkaloids (**Figures 5**, **6**). The results also highlight the convergence of multiple biosynthetic pathways including the shikimic acid/phenylpropanoid pathway, flavonoids, acetate to pentaketide pathway, and L-lysine degradation pathway in the biosynthesis of complex chromone alkaloids like rohitukine. These results form a base for the further comprehensive investigation of the chromone alkaloid biosynthesis that is required for engineering heterologous hosts to make these valuable molecules and their derivatives.

## 4 Conclusions

*D. binectariferum*, an endemic medicinal plant of the Western Ghats, India, is well known to produce a chromone alkaloid called rohitukine and as well as a variety of triterpenoids and flavonoids. Rohitukine is a natural precursor for the semi-synthetic of anticancer drugs flavopiridol and P-276-00. To understand the biosynthetic pathway of rohitukine, we generated a comprehensive transcriptome assembly of leaf and root tissues and identified 51,598 non-redundant protein sequences of more than 200 amino acids. About 78.95% of these unigenes were annotated to the Nr database highlighting the completeness of the assembly. Next, with a combination of metabolite profiling and transcriptome assembly, we presented a biosynthetic route to these diverse compounds including terpenoids, flavonoids, and chromone alkaloids. More specifically, we discover candidate genes in rohitukine biosynthesis, and these enzymes strongly suggest the possibility of involvement of noreugenin pathway in the production of rohitukine and these biosynthetic routes have not been described previously. Therefore, these results pave the way for further functional characterization of these genes and clarify the biosynthesis pathway of chromone alkaloids, specifically rohitukine in *D. binectariferum*.

## Data availability statement

The data presented in the study are deposited found in the NCBI repository, BioProject-PRJNA943416, and SRA accession numbers, SRR23901936 and SRR23901937.

## Author contributions

PK and RT designed the research. PK and EV carried out experiments and analyzed the data. EV, JS and AM participated in sample collection and carried out the qRT-PCR assays. PK, RT and EV wrote the manuscript. The authors read and approved the final manuscript.

## Supplementary material

Additional File 1 Functional annotation of final transcriptome of *D. binectariferum* with different databases.

Additional File 2 Annotated (17 selected) KEGG metabolite pathways of *D. binectariferum.*

Additional File 3 Differential expression of unigenes in leaves and roots of *D. binectariferum.*

Additional File 4 Differential expression of unigenes (DEGs) in root and leaf tissues of *D*. *binectariferum* of selected 17 specialized metabolic pathways.

## Glossary

**Table:**

CDK	Cyclin-dependent kinase
CLL	Chronic Lymphocytic Leukemia
DESI MS	Desorption Electrospray Ionization Mass Spectrometry Imaging
ESI	Electrospray Ionization
LC	Liquid Chromatography
HPLC	High-performance liquid chromatography
RP-18	Reversed Phase –
	
MeOH	Methanol
GC-MS	Gas chromatography- Mass Spectrometry
NIST	National Institute of Standards and Technology
RIN	RNA integrity number
NCBI	National Center for Biotechnology Information
Nr	Non-redundant
Pfam	Protein family
GO	Gene Ontology
COGs	Clusters of Orthologous Groups of proteins
KEGG	Kyoto Encyclopaedia of Genes and Genomes
DEGs	Differentially expressed genes
TFs	Transcription factors
DEGs	Differentially expressed genes
FDR	False-discovery rate
FPKM	Fragments per kilobase per million mapped
KO	KEGG Orthology
EF2	Elongation factor 2
MVA	Mevalonic acid
MEP	2-C-methyl-D-erythritol 4-phosphate
IPP	Isopentenyl diphosphate
DPP	Dimethylallyl diphosphate
GPP	Geranyl diphosphate
FPP	Farnesyl diphosphate
HDR	HDR-4-Hydroxy-3-methylbut-2-enyl diphosphate reductase (HDR)/HMBPP reductase
IDI	IPP isomerase
K12503	short-chain Z-isoprenyl diphosphate synthase
AFS 1	α-farnesene synthase
GGPP	Geranylgeranyl diphosphate
SQLE	Squalene epoxidase
ERG1-	Squalene monooxygenase
FDPS	Farnesyl diphosphate synthase
GERD	(-)-Germacrene D synthase
GA2ox	Gibberellin 2 beta-dioxygenase
KAO	Ent-kaurenoic acid monooxygenase
PAL	Phenylalanine ammonia-lyase
C4H	Trans-cinnamate 4-hydroxylase
4CL	4-coumarate-CoA ligase
CHS	Chalcone synthase
CHI	Chalcone isomerase
DFR	Dihydroflavonol 4-reductase/flavanone 4-reductase
F3H	Flavanone 3-hydroxylase
LAR	Leucoanthocyanidin reductase
ANS	Anthocyanidin synthase
ANR	Anthocyanidin reductase
ldc-C	Lysine decarboxylase
PatA	Putrescine aminotransferase
LYS1 L	Lysine-forming saccharopine dehydrogenase
AASS	Alpha-aminoadipic semialdehyde synthase
LYS9	L-glutamate-forming saccharopine dehydrogenase
PIPOX	Sarcosine oxidase/L-pipecolate oxidase
PCS	Pentaketide chromone synthase
PKS-III	Type III Polyketide synthase.
