# Supplementary material for: De novo transcriptome analysis of Dysoxylum binectariferum to unravel the biosynthesis of pharmaceutically relevant specialized metabolites
Source: Front Plant Sci. 2023 Aug 9;14:1098987. doi: 10.3389/fpls.2023.1098987 (PMC10450223; doi:10.3389/fpls.2023.1098987)
Supplement: Supplementary file 5 [file Presentation_1.pptx]

## Slide 1
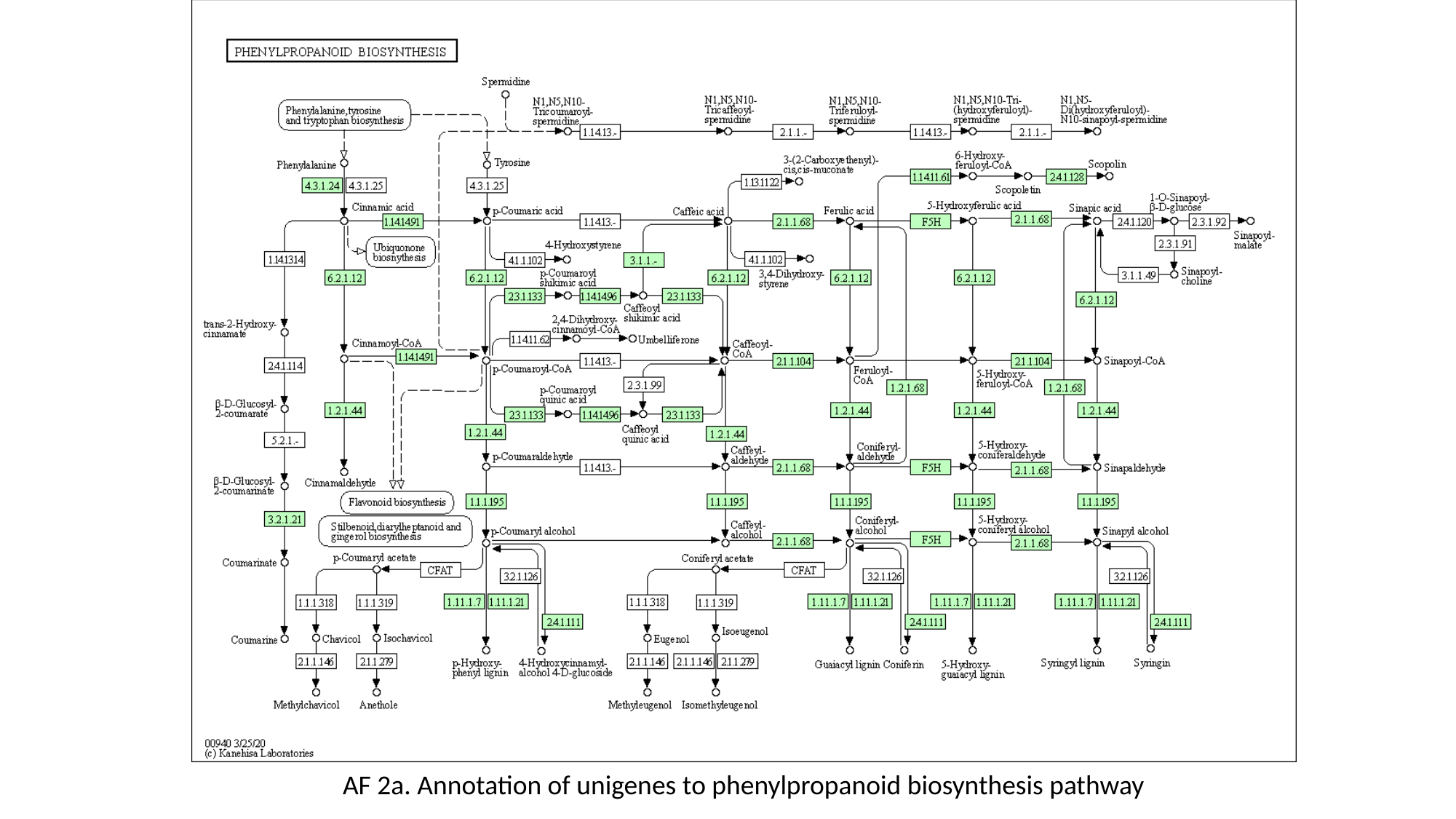

AF 2a. Annotation of unigenes to phenylpropanoid biosynthesis pathway

## Slide 2
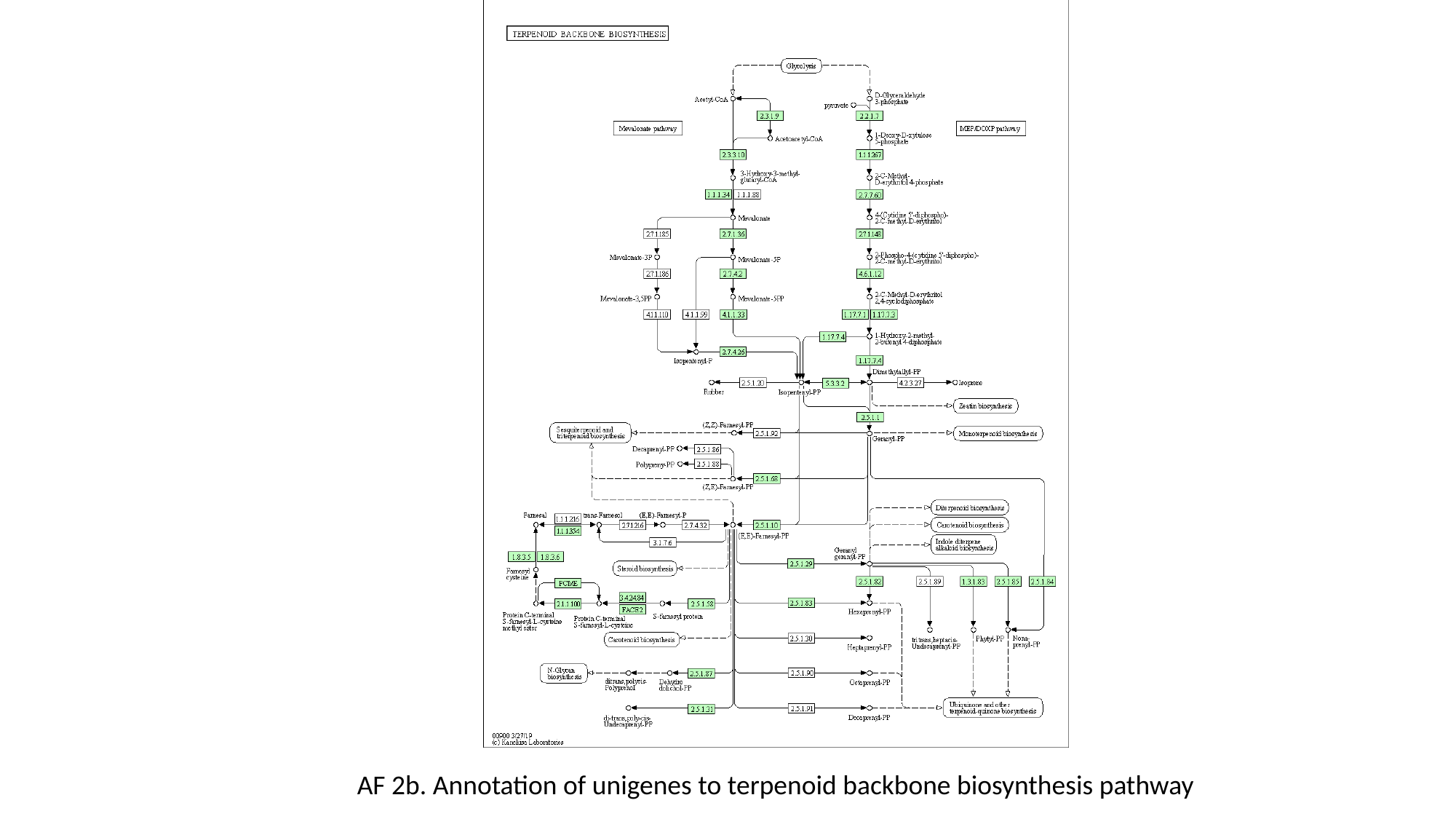

AF 2b. Annotation of unigenes to terpenoid backbone biosynthesis pathway

## Slide 3
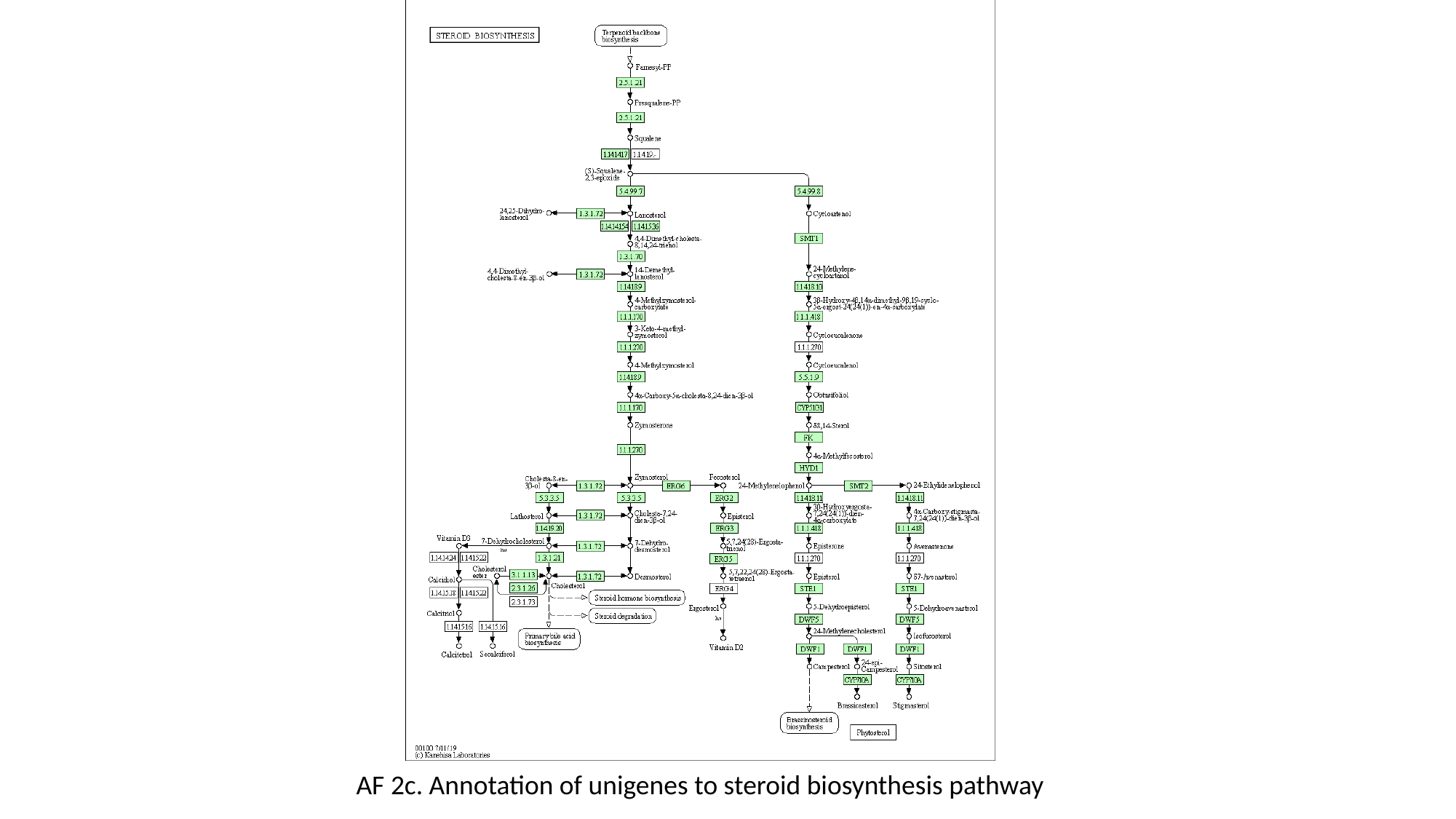

AF 2c. Annotation of unigenes to steroid biosynthesis pathway

## Slide 4
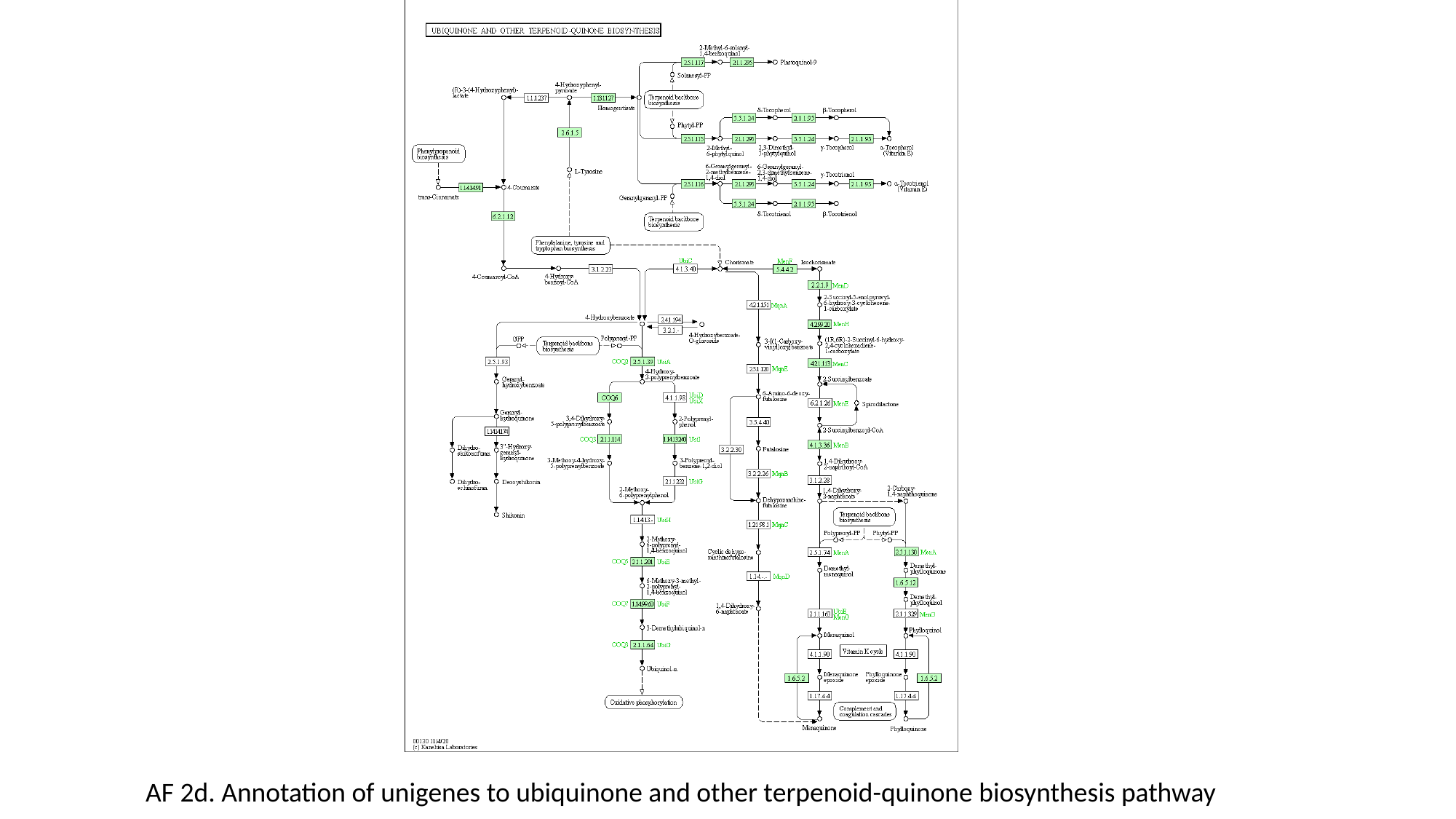

AF 2d. Annotation of unigenes to ubiquinone and other terpenoid-quinone biosynthesis pathway

## Slide 5
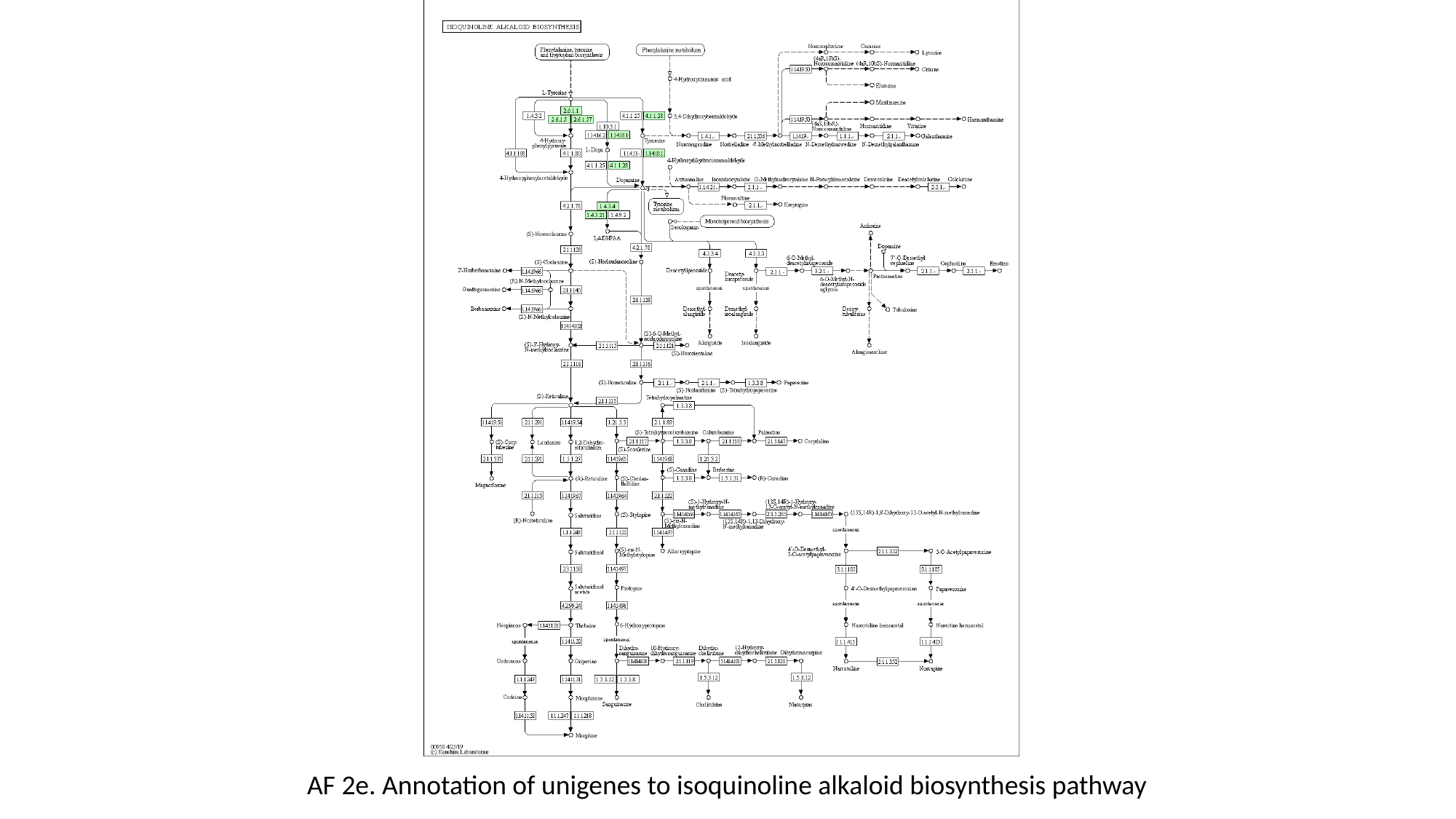

AF 2e. Annotation of unigenes to isoquinoline alkaloid biosynthesis pathway

## Slide 6
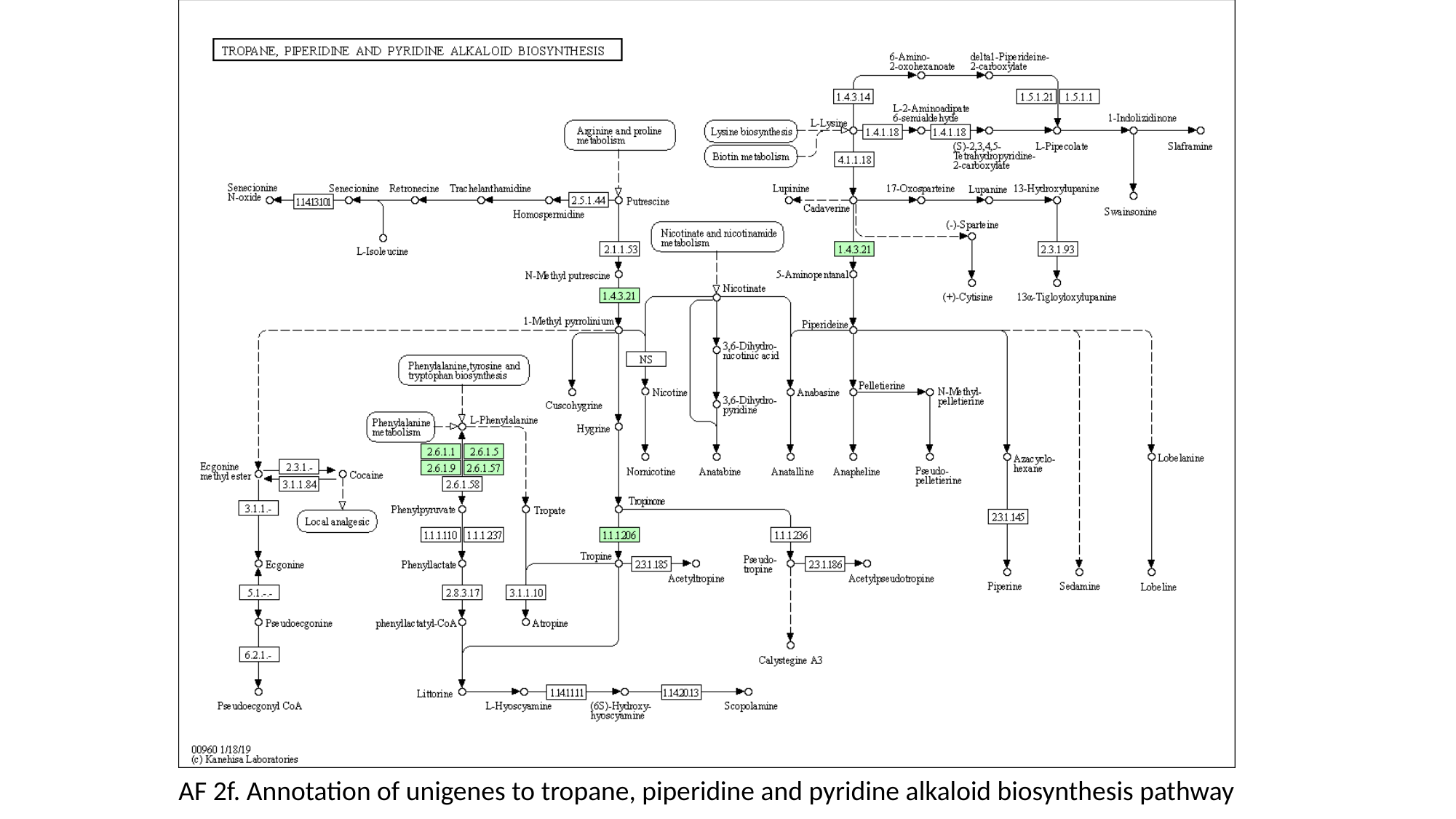

AF 2f. Annotation of unigenes to tropane, piperidine and pyridine alkaloid biosynthesis pathway

## Slide 7
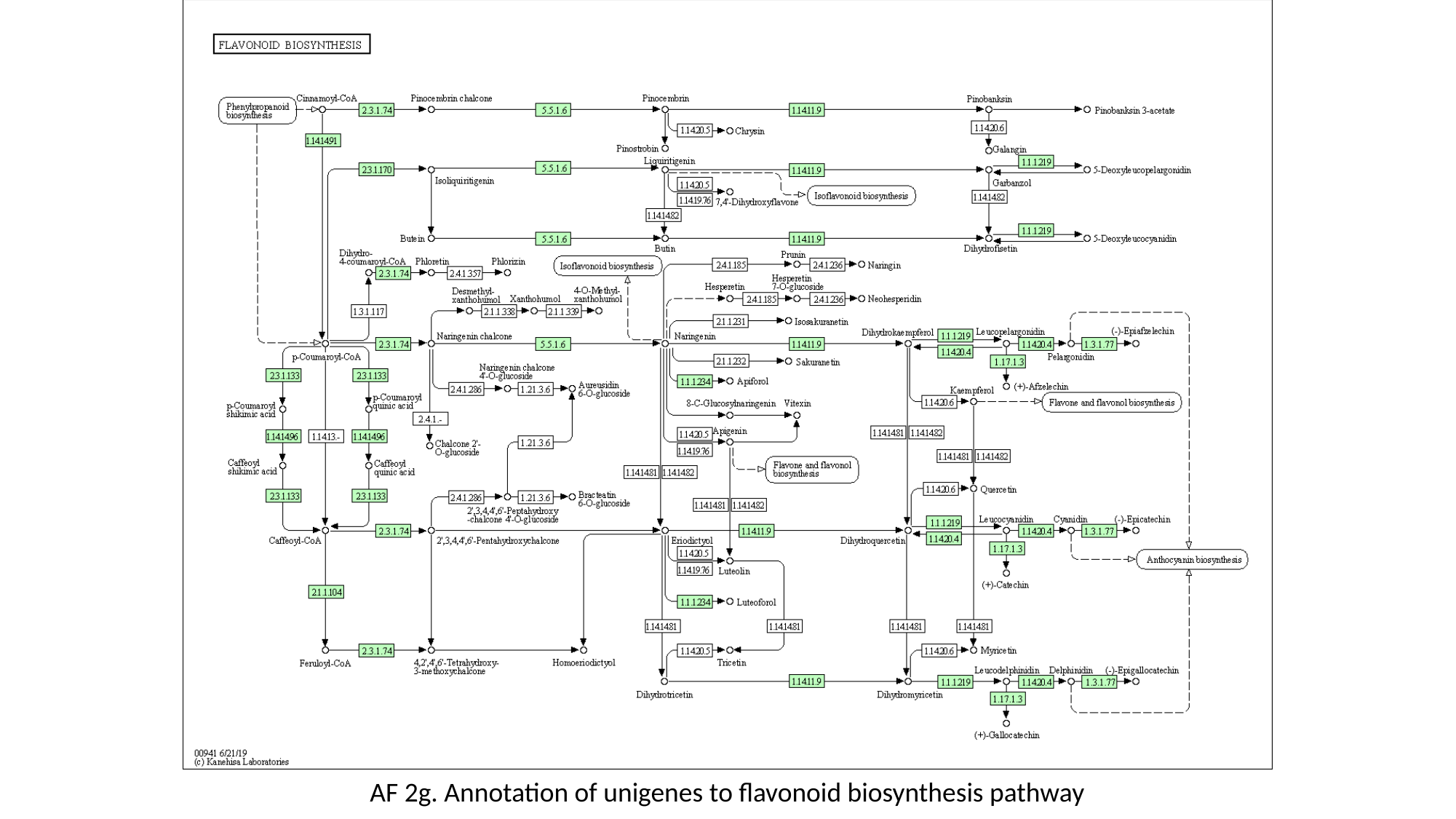

AF 2g. Annotation of unigenes to flavonoid biosynthesis pathway

## Slide 8
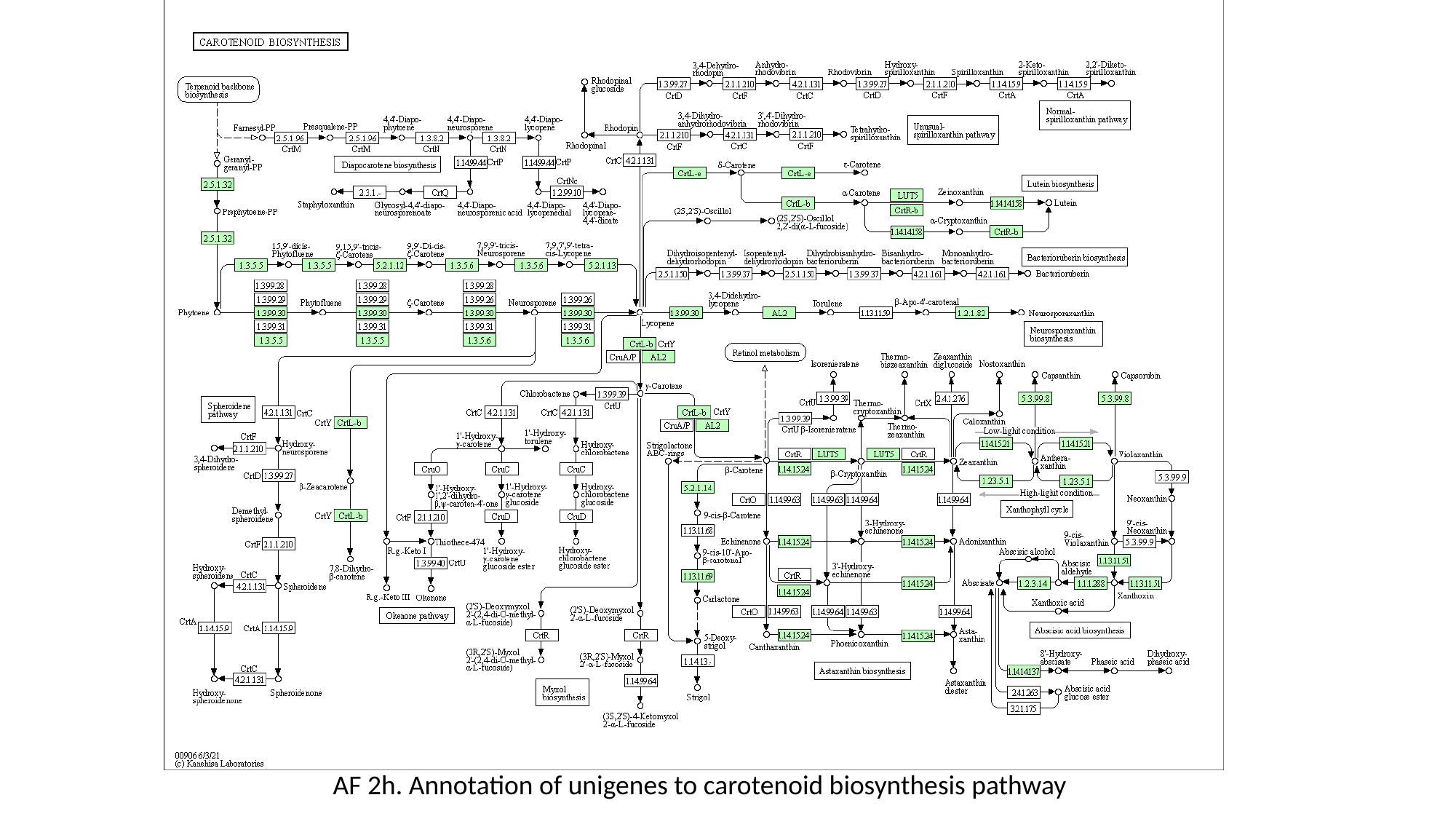

AF 2h. Annotation of unigenes to carotenoid biosynthesis pathway

## Slide 9
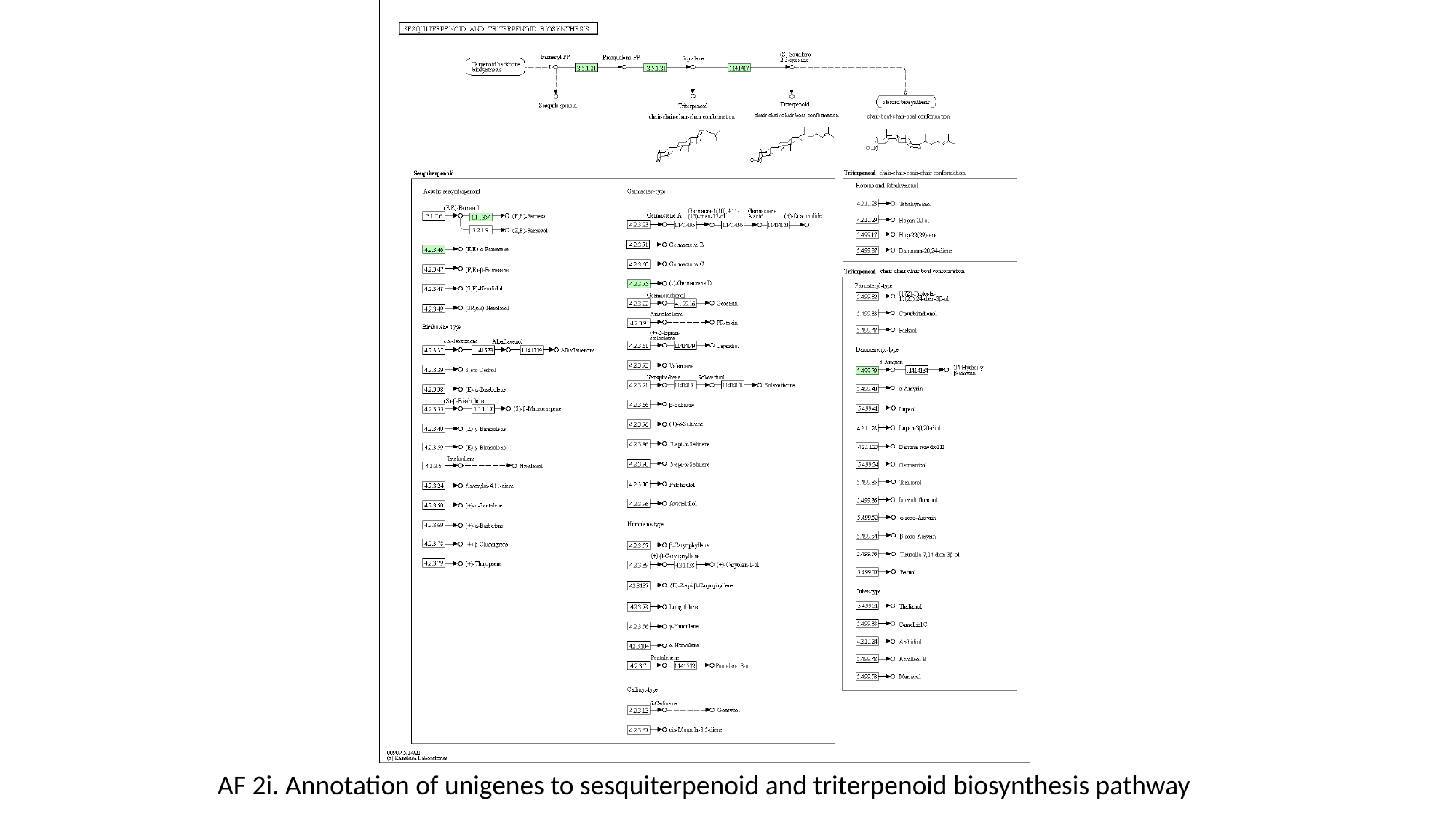

AF 2i. Annotation of unigenes to sesquiterpenoid and triterpenoid biosynthesis pathway

## Slide 10
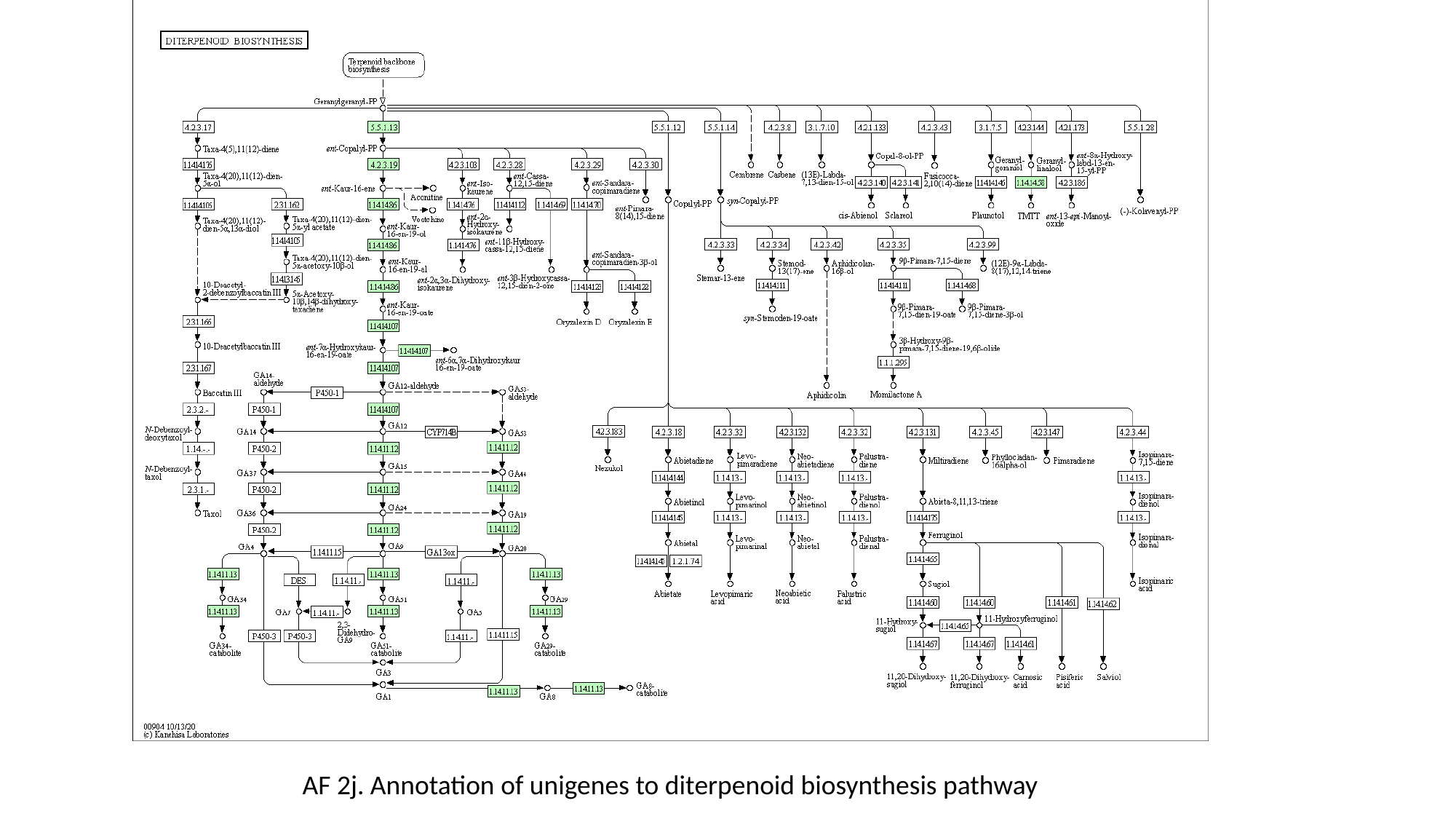

AF 2j. Annotation of unigenes to diterpenoid biosynthesis pathway

## Slide 11
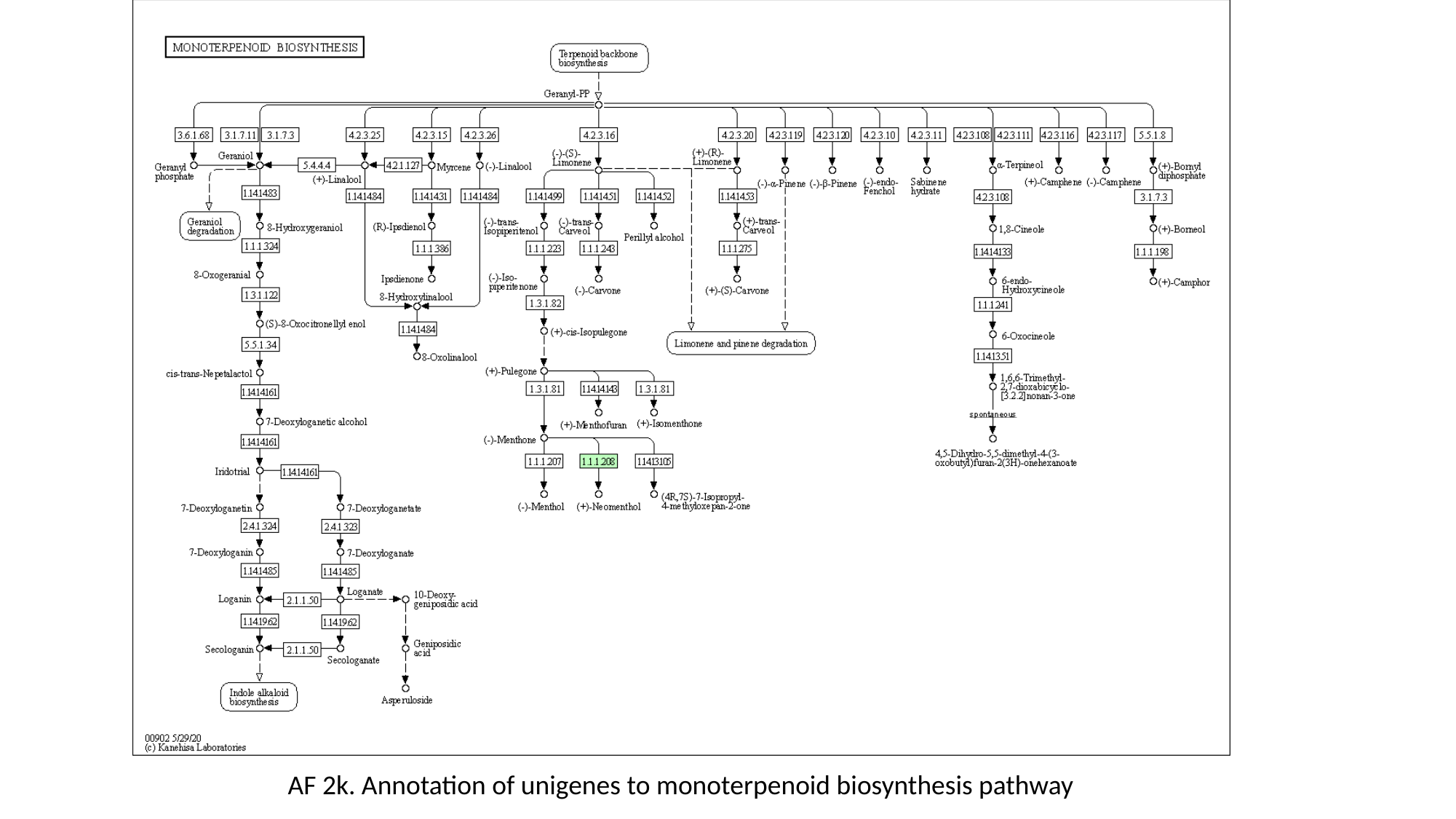

AF 2k. Annotation of unigenes to monoterpenoid biosynthesis pathway

## Slide 12
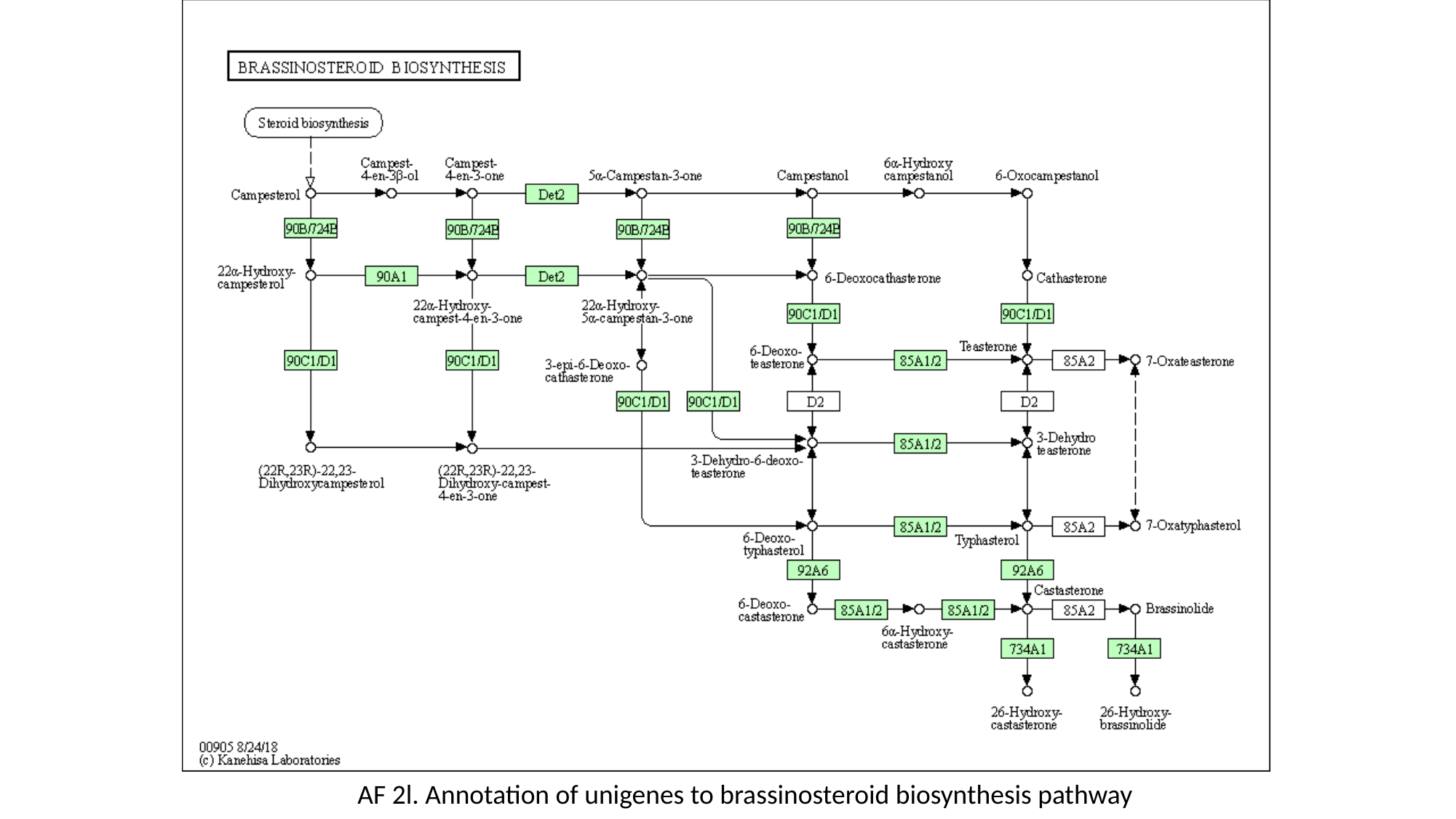

AF 2l. Annotation of unigenes to brassinosteroid biosynthesis pathway

## Slide 13
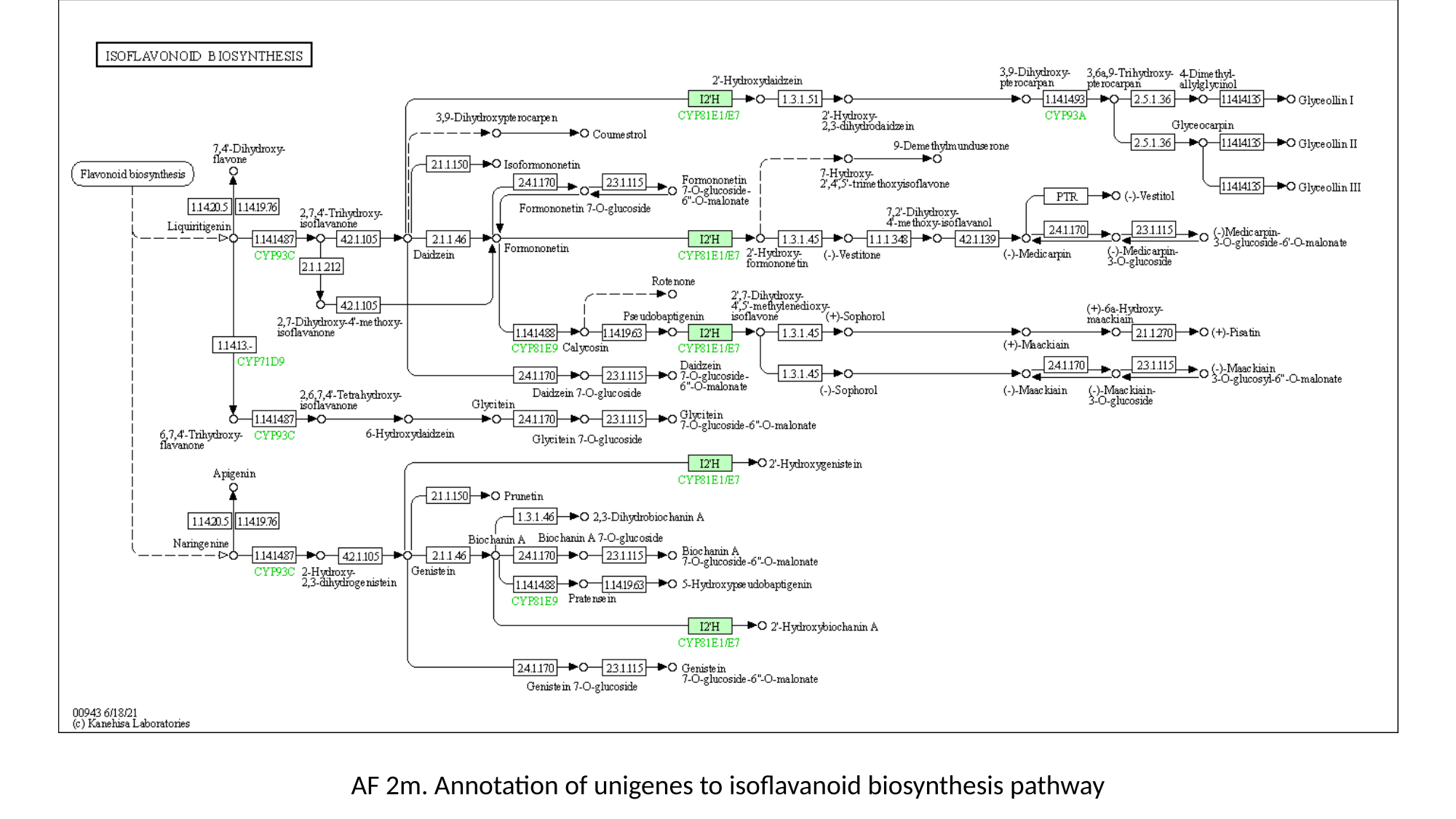

AF 2m. Annotation of unigenes to isoflavanoid biosynthesis pathway

## Slide 14
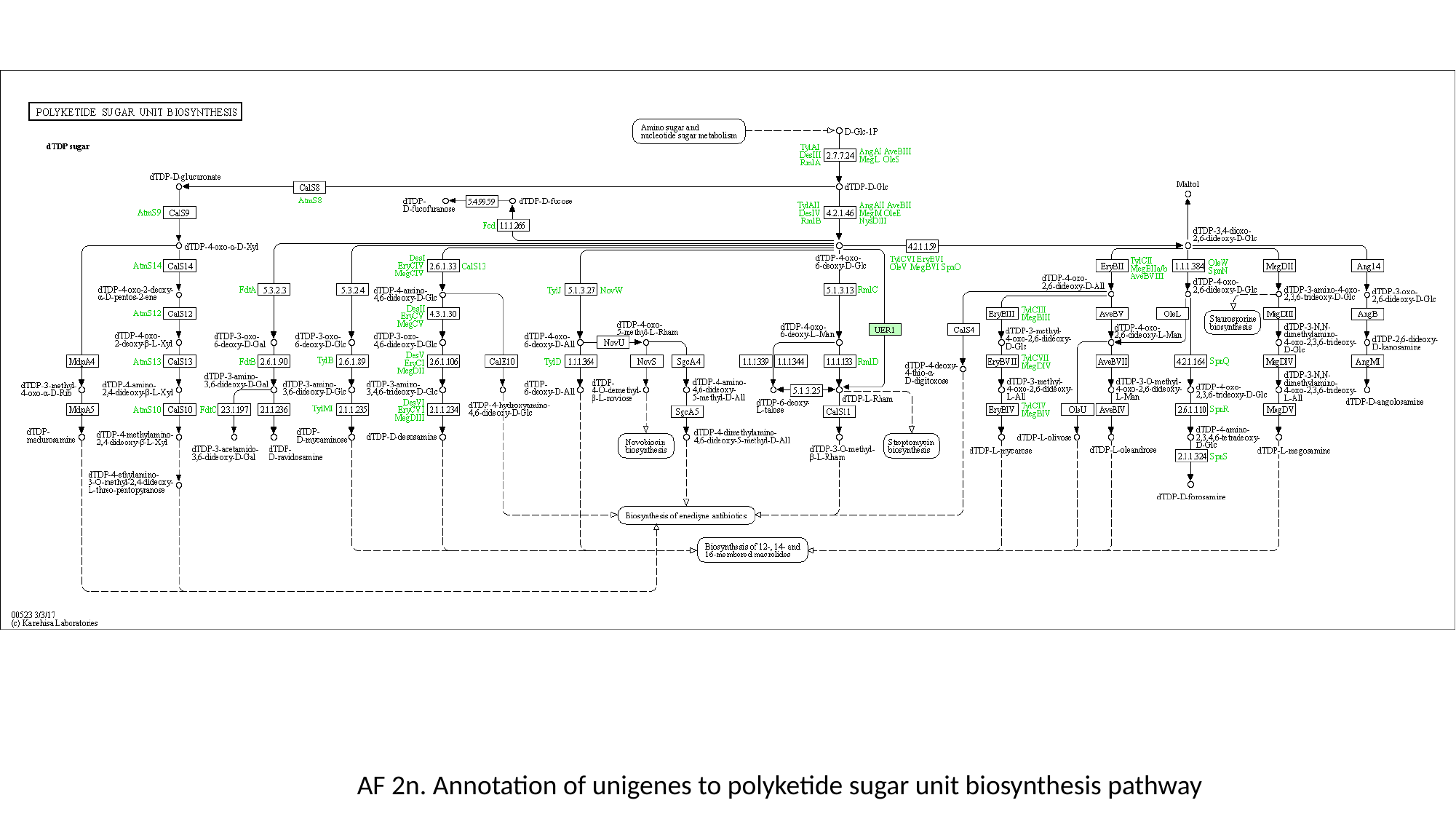

AF 2n. Annotation of unigenes to polyketide sugar unit biosynthesis pathway

## Slide 15
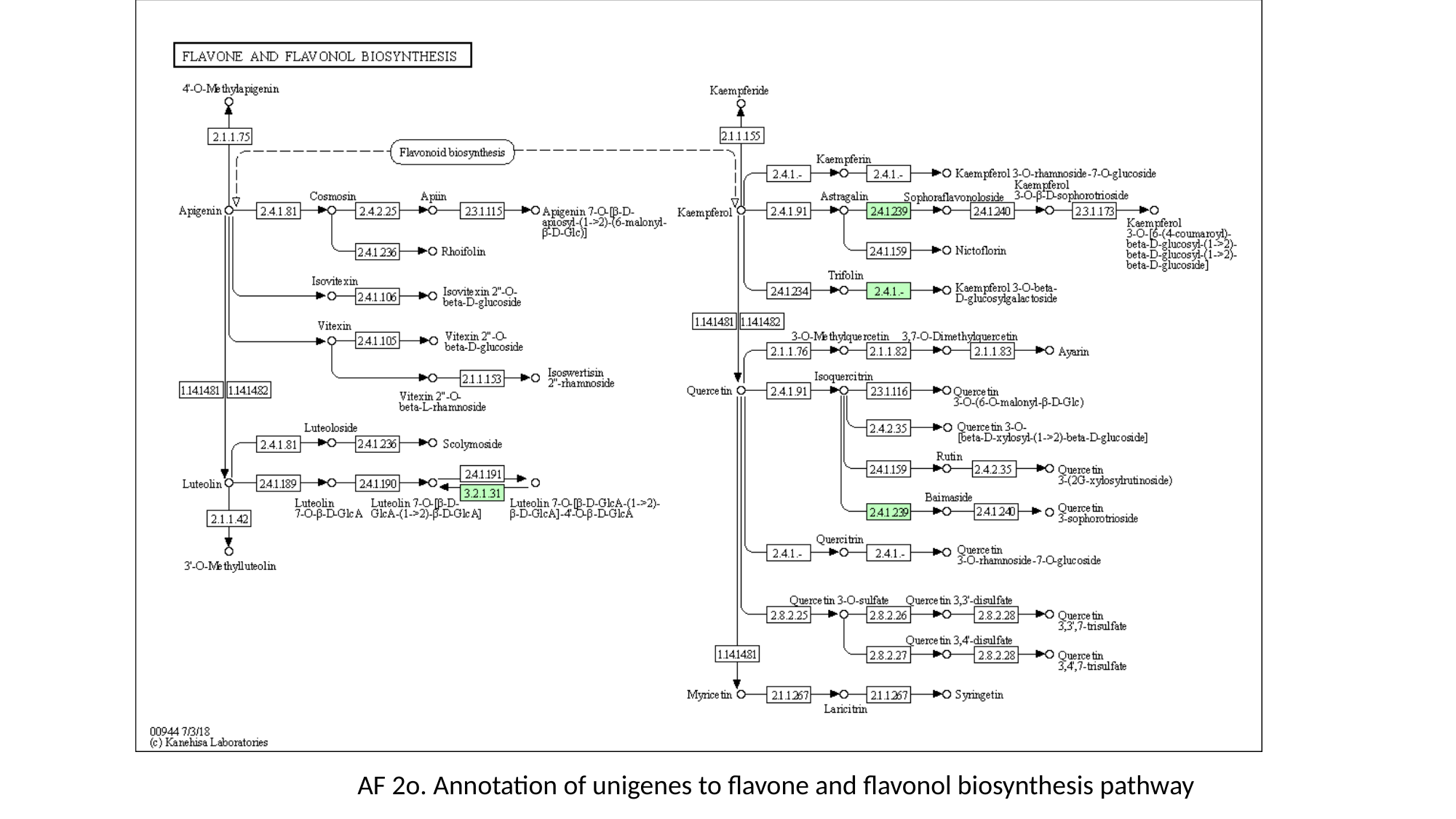

AF 2o. Annotation of unigenes to flavone and flavonol biosynthesis pathway

## Slide 16
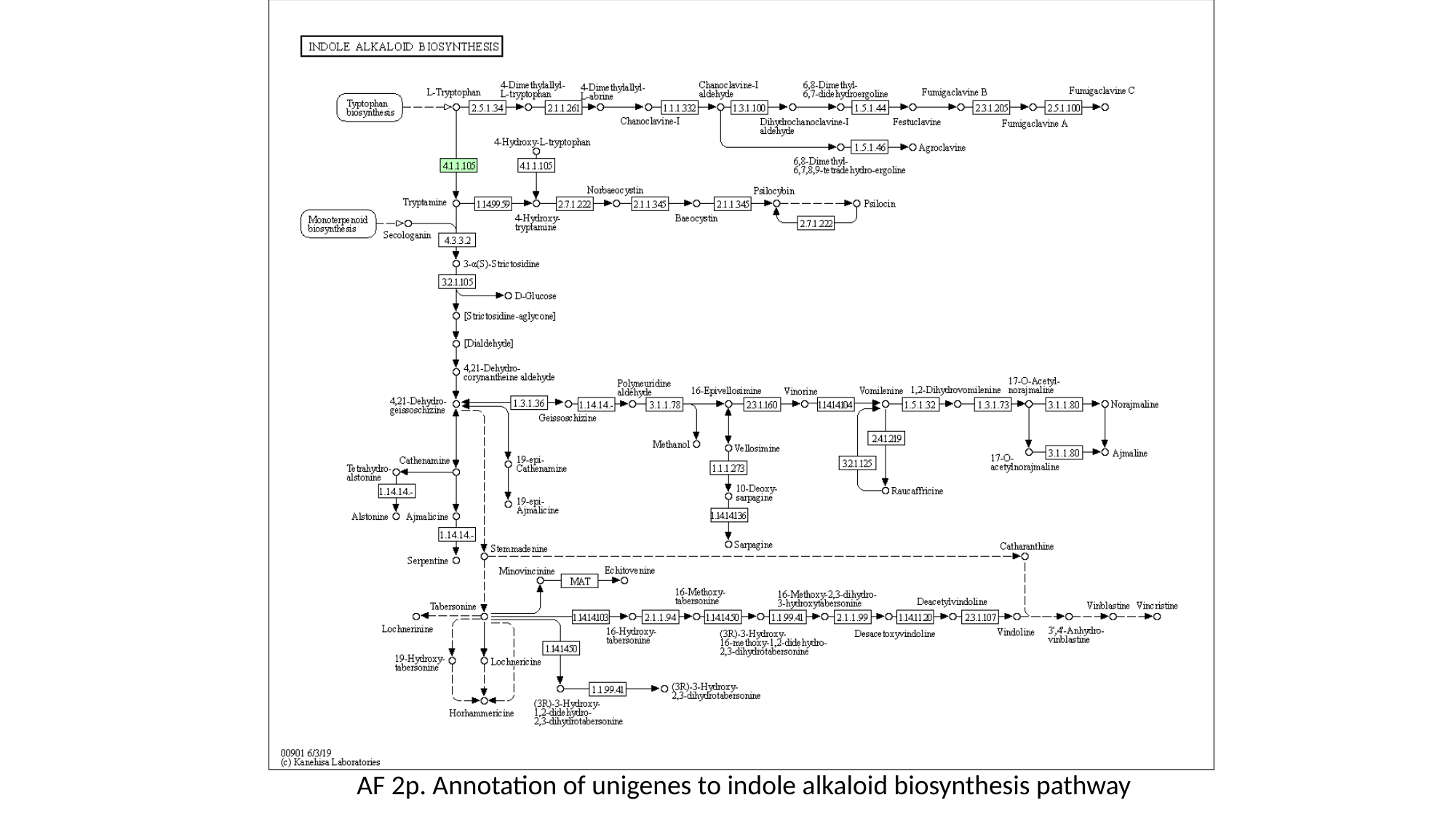

AF 2p. Annotation of unigenes to indole alkaloid biosynthesis pathway

## Slide 17
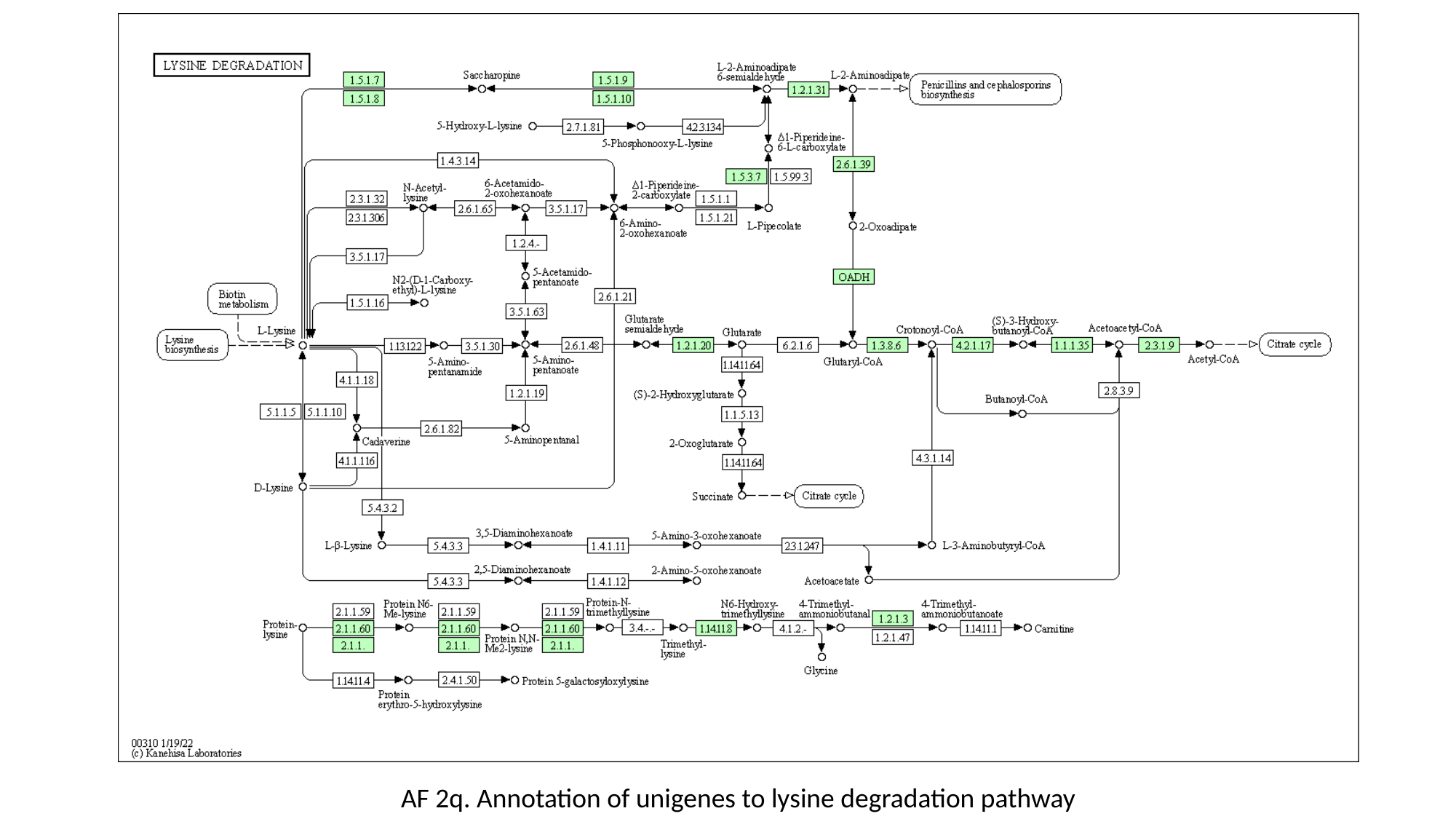

AF 2q. Annotation of unigenes to lysine degradation pathway
