# Supplementary material for: De novo transcriptome analysis of Dysoxylum binectariferum to unravel the biosynthesis of pharmaceutically relevant specialized metabolites
Source: Front Plant Sci. 2023 Aug 9;14:1098987. doi: 10.3389/fpls.2023.1098987 (PMC10450223; doi:10.3389/fpls.2023.1098987)
Supplement: Supplementary file 8 [file Table_3.docx]

**Figure S1.** Summary figure showing overall bioinformatic analysis and target metabolic pathways prediction in *Dysoxylum binectariferum*

**Figure S2.** *D. binectariferum* root and leaf unigenes and their expression profile of MEP and MVP pathways.

**MVP pathway**: AACT, acetoacetyl-CoA thiolase; HMGS, 3-hydroxy-3-methylglutaryl-CoA synthase; HMGR, 3-hydroxy-3-methylglutaryl-CoA reductase; MVK (Mevalonic Acid ), pMVK (Phosphomevalonate Kinase), MDC (Mevalonate-5-phosphate decarboxylase).

**MEP pathway:** DXS, 1-deoxy-D-xylulose-5-phosphate synthase; DXR, 1-deoxy-D-xylulose-5-phosphate reductoisomerase; MCT, 4-diphosphocytidyl-2C-methyl-D-erythritol 4-phosphate synthase; CMK, 4-(cytidine-5′-diphospho)-2-C-methyl-D-erythritol kinase; MDS, 2C-methyl-D-erythritol 2,4-cyclodiphosphate synthase; HDS, 4-hydroxy-3-methylbut-2-enyl diphosphate synthase; HDR, 1-hydroxy-2-methyl-2-(E)-butenyl 4-diphosphate reductase; IDI,Isopentenyl diphosphate isomerase.

a)


b)

**Figure S3.** GCMS chromatogram showing various volatiles from leaf (a) and root (b) of *Dysoxylum binectariferum*


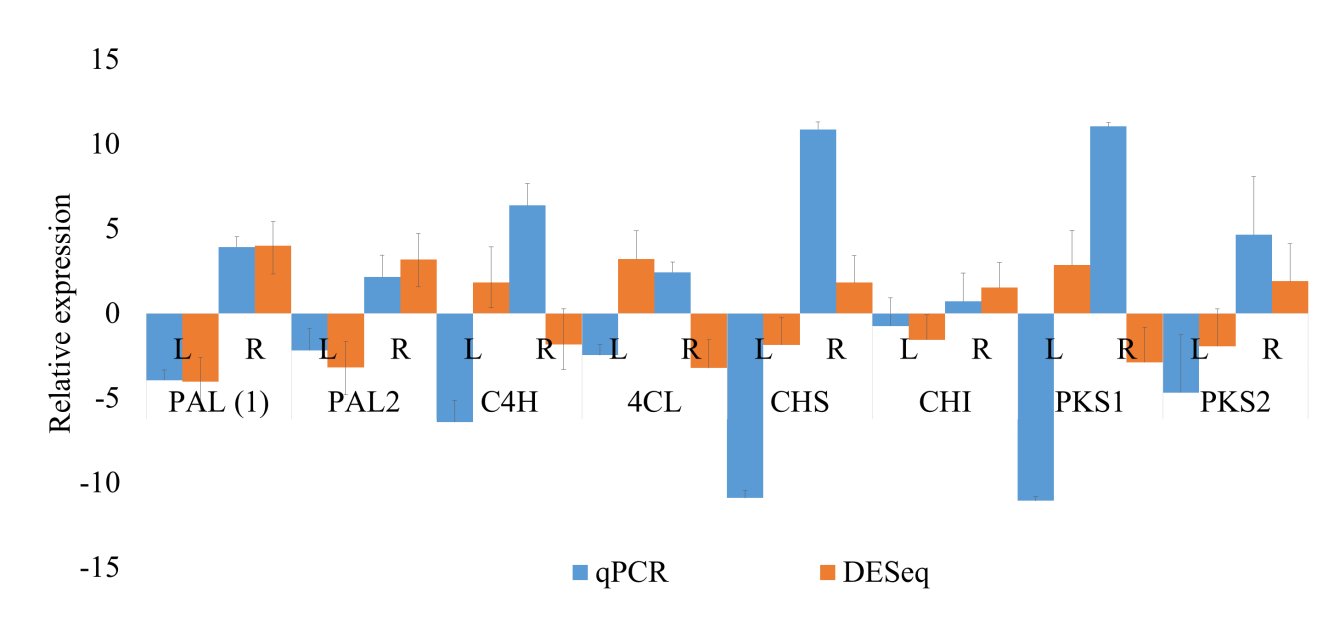


**Figure S4.** Expression patterns (log2fold change) of phenylalanine ammonia-lyase (PAL), trans-cinnamate 4-hydroxylase (C4H), (C4H), 4-coumarate-CoA ligase (4CL), chalcone synthase (CHS), chalcone isomerase (CHI) and type III polyketide synth (PKS-III) candidate genes involved in flavonoid and chromone alkaloid biosynthesis in root and leaf tissues of *D. binectariferum*.
