# Supplementary material for: De novo transcriptome analysis of Dysoxylum binectariferum to unravel the biosynthesis of pharmaceutically relevant specialized metabolites
Source: Front Plant Sci. 2023 Aug 9;14:1098987. doi: 10.3389/fpls.2023.1098987 (PMC10450223; doi:10.3389/fpls.2023.1098987)
Supplement: Supplementary file 9 [file Table_4.docx]

**Table S1** Summary of *Dysoxylum binectariferum* whole transcriptome assembly.

| Assembly | Contigs / Unigenes |
| --- | --- |
| # contigs (>= 0 bp) | 1,127,960 |
| # contigs (>= 1000 bp) | 1,26,788 |
| Total length (>= 0 bp) | 591,008,165 |
| Total length (>= 1000 bp) | 244,883,226 |
| # contigs | 274,970 |
| Largest contig | 48,974 |
| Total length | 346,683,843 |
| GC (%) | 42.6 |
| N50 | 1,560 |
| N75 | 900 |
| L50 | 69,412 |
| L75 | 1,42,746 |
| # N's per 100 kbp | 0 |
| TransDecoder protein | 117,619 |
| CD-HIT PROTEIN (>200) | 51,598 |

**Table S2.** BUSCO analysis of *Dysoxylum binectariferum* transcript assembly

| **Parameter** | **BUSCO (%)**  **Whole transcript** | **BUSCO (%)** Protein |
| --- | --- | --- |
| Lineage: Eudicots_odb10 |  |  |
| Complete BUSCOs (C) | 2142 (92.1%) | 1751 (75.3%) |
| Complete and single-copy BUSCOs (S) | 373 (16.0%) | 1570 (67.5%) |
| Complete and duplicated BUSCOs (D) | 1769 (76.1 %) | 181 (7.8%) |
| Fragmented BUSCOs (F) | 75 (3.2%) | 188 (8.1% |
| Missing BUSCOs (M) | 109 (4.7 %) | 387 (16.6%) |
| Total BUSCO groups searched | 2326 (100%) | |

**Table S3.** Volatile components identified from leaf and root of *D. binectariferum* using GC-MS

| **Sl. No.** | **RT** | **Compounds** | **% rel. area (leaf)** | **% rel. area (root)** | **Class of compound** |
| --- | --- | --- | --- | --- | --- |
| 1 | 3.525 | Cyclohexane, methyl | 14.29 | 0 | Unsaturated aliphatic hydrocarbons |
| 2 | 6.19 | 2-Hexenal | 2.09 | 0.22 | Aldehydes |
| 4 | 9.61 | 5-Hepten-2-one, 6-methyl- | 1.5 | 0.11 | Ketone |
| 6 | 12.99 | Elixene | 1.27 | 0 | Cycloalkanes |
| 8 | 20.735 | (+)-4-Carene | 0.22 | 0 | Monoterpene |
| 9 | 21.265 | α-cubebene | 0 | 3.07 | Sesquiterpene |
| 10 | 21.835 | α-ylangene | 0.76 | 7.64 | Sesquiterpenoid |
| 11 | 21.95 | α-Copaene | 0.57 | 2.03 | Sesquiterpene |
| 13 | 22.325 | (-)-.β.-Bourbonene | 1.39 | 0 | Sesquiterpenoid |
| 14 | 23.29 | Caryophyllene | 0.46 | 0 | Sesquiterpenoid |
| 15 | 23.42 | β-copaene | 0.34 | 5.82 | Sesquiterpene |
| 16 | 23.69 | Alloaromadendrene | 1.82 | 0 | Sesquiterpenoid |
| 17 | 24.25 | α-Guaiene | 0.83 | 0 | Sesquiterpenoid |
| 18 | 24.39 | Germacrene D | 0.63 | 0 | Sesquiterpenoid |
| 19 | 24.825 | α-Muurolene | 0.99 | 36.78 | Sesquiterpene |
| 20 | 25.21 | γ-Cadinene | 1.01 | 0 | Sesquiterpene |
| 21 | 25.28 | δ-Cadinene | 3.67 | 0 | Sesquiterpene |
| 22 | 25.562 | Cedrene | 0 | 2.05 | Sesquiterpene |
| 23 | 26.705 | Spathulenol | 0.63 | 0 | Sesquiterpenoid |
| 24 | 29.263 | α-cadinol | 0 | 1.22 | Sesquiterpenoid |

**Table S4.** Primer details of the selected genes used for the RT PCR validation

| **Name** | **Transcript ID** | **Primer Sequence 5’-3’** | **Product size** |
| --- | --- | --- | --- |
| CHS | TRINITY_DN149243_c0_g1_i3.p1 | F: CGTGTGCTCTGAGATAACTG | 120 |
|  |  | R: GGATCAGCACCGATGATAAG |  |
| PKS (1) | TRINITY_DN1192_c0_g1_i12.p1 | F: GATCTGTTGCCAGAGAAACT | 108 |
|  |  | R: GCGCTCTTCTATCATGTACTC |  |
| PKS (2) | TRINITY_DN4064_c0_g1_i12.p1 | F: CTTCGCCCGTCTGTTAAA | 112 |
|  |  | R: GAACCCGAGCACCTTTATT |  |
| 4CL | TRINITY_DN51831_c0_g1_i4.p1 | F: GTAGACGGAGAGAACCCAAATC | 116 |
|  |  | R:CCAGCTCTGAGGGAACATAAG |  |
| PAL(1) | TRINITY_DN2548_c0_g1_i11.p1 | F:CTCGATGAGGTGAAACGTATGG | 120 |
|  |  | R: TTGCTGTGACTTGGCCTATG |  |
| PAL(2) | TRINITY_DN207337_c1_g1_i3.p1 | F:ACTCCTTGGGACTGATCTCTT | 128 |
|  |  | R: TCTCCTCCAAATGCCTCAAATC |  |
| CHI | TRINITY_DN2210_c0_g1_i1.p1 | F: CGTTAACCGGTGCACAATAC | 126 |
|  |  | R: CGTCCTTGAAGACCTCGATAAA |  |
| C4H | TRINITY_DN93492_c0_g1_i2.p1 | F:GAGGTCTCCAGCCATTAATATCC | 114 |
|  |  | R: CCCACCATCTTCTTCCTTTCTT |  |

| **Table S5:** Selected gene contig corresponds to the sequence homology/ similarity identity with reported genes in the NCBI database | | | | | | |  |
| --- | --- | --- | --- | --- | --- | --- | --- |
| **Sl. No.** | **Gene** | **Contig** | **Homologous Contig** | **Genes** | **Species** | **% Identity** | **% Coverage** |
| 1 | PKS | TRINITY_DN1192_c0_g1_i12.p1 | RVW93317.1 | Type III PKS B | *Vitis vinifera* | 83.63 | 97 |
|  |  |  | KAH9696797.1 | Type III PKS B | *Citrus sinensis* | 83.72 | 97 |
|  |  |  | sp\|Q8LDM2\|PKSB_ARATH | Type III PKS B | *Arabidopsis thaliana* | 81.14 | 97 |
|  |  |  | sp\|O23674\|PKSA_ARATH | Type III PKS A | *Arabidopsis thaliana* | 66.06 | 97 |
|  |  |  | sp\|C4NF91\|PKS5_ALOAR | PKS5 | *Aloe arborescens* | 38.78 | 95 |
|  |  |  | sp\|C4MBZ5\|PKS3_ALOAR | PKS3 | *A. arborescens* | 37.69 | 96 |
|  |  |  | sp\|Q58VP7\|PCS_ALOAR | PCS | *A. arborescens* | 37.44 | 96 |
|  |  |  | sp\|Q3L7F5\|OKS_ALOAR | OKS | *A. arborescens* | 37.44 | 95 |
|  |  |  | sp\|C4NF90\|PKS4_ALOAR | PKS4 | *A. arborescens* | 37.44 | 95 |
| 2 | CHS | TRINITY_DN149243_c0_g1_i3.p1 | KAJ4722929.1 | Chalcone synthase | *Melia azedarach* | 95.9 | 99 |
|  |  |  | NP_001313780.1 | Chalcone synthase 1-like | *Gossypium hirsutum* | 94.09 | 98 |
|  |  |  | AGH13332.1 | Chalcone synthase | *Rhus chinensis* | 93.85 | 99 |
|  |  |  | sp\|P48388.1\|CHS3_CAMSI | Chalcone synthase 3 | *Camellia sinensis* | 92.54 | 99 |
| 3 | CHI | TRINITY_DN61855_c0_g1_i2.p1 | XP_006435919.1 | Probable chalcone--flavonone isomerase 3 | *Citrus clementina* | 88.18 | 97 |
|  |  |  | KAH9702872.1 | Putative chalcone--flavonone isomerase 3 | *Citrus sinensis* | 88.18 | 97 |
|  |  |  | KAH9767501.1 | Putative chalcone--flavonone isomerase 3 | *Citrus sinensis* | 87.68 | 97 |
|  |  |  | AUT30534.1 | chalcone isomerase | *Camellia fraterna* | 81.28 | 97 |
| 4 | PAL | TRINITY_DN2548_c0_g1_i11.p1 | KAJ4720771.1 | Phenylalanine ammonia-lyase | *Melia azedarach* | 92.57 | 99 |
|  |  |  | XP_031257444.1 | Phenylalanine ammonia-lyase-like | *Pistacia vera* | 89.96 | 99 |
|  |  |  | KAJ4828218.1 | Phenylalanine ammonia-lyase | *Turnera subulata* | 88.64 | 99 |
|  |  | TRINITY_DN8221_c0_g2_i3.p1 | KAJ4726068.1 | Phenylalanine ammonia-lyase | *M. azedarach* | 86.31 | 96 |
|  |  |  | KAH9700509.1 | Phenylalanine ammonia-lyase 1 | *C. sinensis* | 82.8 | 96 |
|  |  |  | KAH9700507.1 | Phenylalanine ammonia-lyase 1 | *C. sinensis* | 82.8 | 96 |
|  |  | TRINITY_DN207337_c1_g1_i3.p1 | NP_001307538.1 | Phenylalanine ammonia-lyase 3 | *Solanum lycopersicum* | 100 | 99 |
|  |  |  | XP_049358909.1 | Phenylalanine ammonia-lyase-like | *Solanum verrucosum* | 98.11 | 99 |
|  |  |  | XP_015087665.1 | phenylalanine ammonia-lyase-like | *Solanum pennellii* | 99.27 | 99 |
| 5 | 4CL | TRINITY_DN2283_c1_g2_i5.p1 | KAJ4702239.1 | 4-coumarate-CoA ligase | *Melia azedarach* | 90.35 | 99 |
|  |  |  | XP_006435318.1 | 4-coumarate--CoA ligase-like 5 isoform X1 | *Citrus clementina* | 85.19 | 99 |
|  |  |  | KAH9693716.1 | 4-coumarate--CoA ligase-like 5 | *Citrus sinensis* | 85.37 | 99 |
|  |  | TRINITY_DN51831_c0_g1_i4.p1 | KAJ4721013.1 | 4-coumarate-CoA ligase | *Melia azedarach* | 87.79 | 98 |
|  |  |  | XP_031248582.1 | 4-coumarate--CoA ligase 2 | *Pistacia vera* | 84.49 | 98 |
|  |  |  | XP_021282810.1 | 4-coumarate--CoA ligase 2 | *Herrania umbratica* | 82.54 | 99 |
